# *Mycobacterium tuberculosis* Modulates the Expansion of Terminally Exhausted CD4^+^ and CD8^+^ T-Cells in Individuals with HIV-TB Co-Infection

**DOI:** 10.3390/pathogens14080802

**Published:** 2025-08-11

**Authors:** Komal Sharma, Aman Sharma, Sunil K. Arora

**Affiliations:** 1 Molecular Immunology Laboratory, Department of Immunopathology, Post Graduate Institute of Medical Education & Research (PGIMER), Chandigarh 160012, India; komalsharma240193@gmail.com; 2 Department of Internal Medicine, Post Graduate Institute of Medical Education & Research (PGIMER), Chandigarh 160012, India

**Keywords:** HIV-TB co-infection, HIV, T-cell exhaustion, HIV disease progression, immune checkpoint molecules (ICMs), PD-1, CTLA-4, TIM-3, LAG-3, TIGIT

## Abstract

Introduction: *Mycobacterium tuberculosis* (Mtb), the most common co-infection among people living with HIV (PLWH), aggravates the associated morbidity and mortality in these individuals; however, the immune-modulatory role of Mtb in the pathogenesis of HIV infection remains incompletely understood. Methods: We investigated the role of Mtb infection in regulating adaptive immune responses with reference to the expression of five immune checkpoint molecules (ICMs) in co-infected individuals in a cross-sectional study conducted on treatment-naïve human cohorts from North India, including PLWH, people with Mtb infection, people with HIV-Mtb co-infection, and healthy volunteers as controls. Results: The data revealed a significantly increased gene expression of TIM-3 (*p *= 0.0058), LAG-3 (*p *< 0.0001), PD-1 (*p *= 0.0090), and CTLA-4 (*p *= 0.0008). It also revealed a higher frequency of CD4^+^ and CD8^+^ T-cells surface-expressing TIM-3^+^, CTLA-4^+^, LAG-3^+^. Finally, it showed cells co-expressing two ICMs together (*p *< 0.05) in individuals with HIV–Mtb co-infection as compared to HIV mono-infected ones. Interestingly, the frequency of these cells correlated inversely with the absolute CD4^+^ T-cell count and positively with the plasma viral load (*p *< 0.05), indicating direct association with HIV disease progression. Conclusions: These findings suggest that Mtb co-infection exacerbates immune exhaustion in co-infected individuals. Targeting ICMs with pharmacological immune checkpoint inhibitors (ICIs) offers a promising approach for better clinical management of co-infected individuals.

## 1. Introduction

Tuberculosis (TB) continues to be one of the leading infectious causes of morbidity and mortality worldwide. According to the World Health Organization (WHO), approximately a quarter of the world population is latently infected with TB, and around 5–10% of people infected with TB eventually progress to active TB disease, particularly if their immune system becomes compromised. In 2023, around 10.8 million people fell ill with TB worldwide, and an estimated 1.25 million people died from TB [1]. Human immunodeficiency virus (HIV) is the causative agent of acquired immunodeficiency syndrome (AIDS), a condition where a severely immune-compromised state, due to decreased CD4^+^ T-lymphocyte population, allows various life-threatening opportunistic infections like Mtb to thrive [1,2]. It has been reported that individuals living with HIV not receiving effective anti-retroviral therapy (ART) have ≥20-fold higher risk of developing active TB than uninfected individuals due to increased reactivation of latent TB or increased susceptibility to Mtb infection. In 2024, around one-third of the 40.8 million [37–45.6 million] people living with HIV/AIDS (PLHA) were co-infected with Mtb. India, with the highest TB burden globally, also has the third highest number of PLHA and high rates of HIV-associated TB [3]. Despite extensive research advancements and improved understandings of HIV and TB individually, their co-infection in a single host create havoc. This situation thus further complicates the clinical scenario, as patient non-compliance, drug interactions, and overlapping toxic effects contribute to the development of TB-associated immune reconstitution inflammatory syndrome (TB-IRIS), resulting in increased mortality rates in co-infected individuals [4]. Our understanding of the possible mechanisms underlying disease deterioration, rapid progression, and higher mortality rates in co-infected individuals remains incomplete.

Immune checkpoint molecules (ICMs) are important regulators of T-cell functions and are necessary for achieving full T-cell activation. Their activation is multifaceted and requires distinct signals. The first signal occurs when the TCR recognizes an antigenic peptide presented by MHC molecules on antigen presenting cells (APCs). The second signal, known as the costimulatory signal for T-cell activation in an infection, can either be positive or negative, with the former being necessary for initiation of effective immunity, while the latter is necessary for the establishment and maintenance of peripheral tolerance and abortive T-cell responses [5,6]. Positive costimulatory molecules include inducible T-cell costimulator (ICOS), OX40 (CD134), 4-1BB (CD137), and many more that promote T-cell function. Inhibitory molecules such as programmed cell death receptor-1 (PD-1), Cytotoxic T-Lymphocyte Antigen-4 (CTLA-4), Lymphocyte Activation Gene-3 (LAG-3), T-cell Immunoglobulin and Mucin-domain containing-3 (TIM-3), and T-cell immunoreceptor with Ig and immunoreceptor tyrosine-based inhibitory motif domains (TIGIT) suppress T-cell activation. A balance between the expressions of these costimulatory/co-inhibitory molecules is necessary to sustain a normal protective response [7,8,9,10]. The upregulated expression of inhibitory molecules in various cancers [11,12,13] and many infectious conditions [14,15,16] is considered a survival mechanism that enables pathogens or tumor cells to evade effective immune responses in a host. Targeting inhibitory ICMs has proven successful in the clinical management of various cancers [17,18,19,20], chronic immune suppression diseases [21,22], and viral infections [23,24]. An upregulated expression of various ICMs including PD-1 [25,26,27], CTLA-4 [28,29], TIM-3 [30,31,32], LAG-3 [33,34], and TIGIT [35,36] has been reported in HIV-1 infection. In vitro blockage of these ICMs has been shown to enhance the proliferative capacity of HIV-specific T-cells and invariant natural killer T (iNKT)-cells [32,36,37,38,39]. Blocking these ICMs has been shown to reverse HIV latency even more effectively than latency-reversing agents [40]. Additionally, it has also been reported that PD-1 blockade, when combined with latency-reversing agents, enhances HIV latency reversal without increasing T-cell activation [41]. Similarly, in Mtb infection, there is an upregulated expression of various ICMs, including PD-1 [42], PD-L1 [43,44,45], TIM-3 [46,47], and LAG-3 [48], on immune cells. In vitro blockade of PD-L1 has been shown to enhance Mtb-specific CD8^+^ T-cell-mediated killing of CD14^+^ cells derived from human tuberculous pleural effusion samples [44]. Conversely, another study reported that PD-1 blockade accelerated Mtb growth via excessive TNF-α secretion [49]. Moreover, blocking TIM-3 restored IFN-γ secretion and improved NK cell-mediated control of Mtb [46], while silencing LAG-3 signaling enhanced Mtb killing by CD4^+^ T-cells [48]. Furthermore, genetic polymorphisms in PDCD1 and TIM-3 have also been associated with increased TB susceptibility in men [50].

Although a few studies on either HIV-TB co-infected individuals or HIV-infected cells with γ-irradiated Mtb have shed light on the expression of different ICMs across various T-cell subsets [51,52,53,54], there is a scarcity of data related to the expression of multiple ICMs in individuals with HIV-TB co-infection, especially in the Indian subcontinent, which bears a significant portion of the global disease burden. Most of the studies on the immunopathogenesis of co-infection have focused on limited innate or cellular immune responses in individuals with co-infection and have been predominantly conducted in regions where HIV-1 subtype B is prevalent [29,40,51,52,54,55,56]. This emphasizes the need for more research efforts from TB endemic countries like India, involving individuals predominantly infected with HIV-1 subtype C with higher prevalence of HIV-TB co-infection and further in-depth comprehension on the possible immune-modulations among these individuals. Thus, we hypothesize that the excessive immune activation due to Mtb co-infection in individuals with HIV-1 infection would have resulted in modulated expression of ICMs on immune cells, leading to accelerated replication of HIV-1, causing faster disease progression and higher mortality rates in these individuals. To test this hypothesis, we assessed the differential expression of five ICMs viz. PD-1, CTLA-4, TIM-3, LAG-3 and, TIGIT on both CD4^+^ and CD8^+^ T-lymphocytes in the peripheral blood of treatment-naïve individuals from four cohorts, including active pulmonary tuberculosis (TB), HIV-1 infection only, HIV-TB co-infected, and healthy volunteers from North India. Furthermore, we have tried to calculate the correlations of immune parameters with HIV disease progression in terms of peripheral blood CD4^+^ T cell count and plasma viral load in these individuals. This study on human subjects from a high-burden state of North India, infected with HIV-1 subtype-C and Mtb, is the first of its kind to reveal the impact of co-infection on expression of these molecules having a significant clinical implication in terms of suggestive use of ICIs as an adjunct therapy for a better management of disease in these individuals.

## 2. Materials and Methods

### 2.1. Study Population

A total of fifty-five human subjects were recruited in the study, categorized into four groups: treatment-naïve individuals living with HIV-1 subtype-C virus only [*n* = 15; age range: 22–44 years]; treatment-naïve individuals co-infected with HIV-1 and Mtb [*n* = 10; age range: 23–55 years]; treatment-naïve individuals infected with Mtb only [*n* = 15; age range: 19–55 years]; and age- and demography-matched healthy human volunteers [*n* = 15; age range: 26–44] as controls. Inclusion criteria for all study groups included treatment-naïve adults aged 18–60 years. Treatment naïve status was defined as individuals who had not received ART or ATT before the time of recruitment and sample collection. This study was conducted at the Post Graduate Institute of Medical Education & Research (PGIMER) attached with a tertiary care hospital, located in the city of Chandigarh in North India. Treatment-naïve individuals infected with HIV-1 subtype-C were recruited from the National AIDS Control Organization (NACO, Ministry of Health and Family Welfare, Govt. of India)-approved Integrated Counselling and Testing Centre (ICTC) and Anti-retroviral treatment (ART) Clinic in the out-patient department of Internal Medicine. The presence of HIV-1 among PLHA was confirmed in the ICTC by NACO-strategy-III as per National guidelines for HIV/AIDS diagnosis [57]. Treatment-naïve individuals infected with Mtb only were recruited from the DOTS (Directly Observed Treatment, Short-course) center under Department of Pulmonary Medicine. The presence of Mtb was detected by sputum smear/culture and nucleic acid amplification test (NAAT). All study participants were screened for co-morbid conditions and individuals diagnosed with Hepatitis B or Hepatitis C infection and/or any other chronic diseases like severe anemia, leukemia, lymphoma, inflammatory disorders, diabetes mellitus, hyperlipidemia, atherosclerosis, coronary artery disease (CAD), chronic kidney disease (CKD), chronic liver diseases (cirrhosis, hepatitis), fatty liver disease, alcoholic liver disease, lung infections (other than TB), neoplasms, intravenous drug users (IDUs) or on treatment for any of these conditions were excluded from the study.

The clinical parameters and the demographic characteristics of subjects recruited in the study are summarized in Table 1. The mean age of individuals co-infected with HIV and Mtb (mean ± SD: 39 ± 10.3) was comparable to that of individuals mono-infected with HIV (mean ± SD: 35.2 ± 6.46), mono-infected with Mtb (mean ± SD: 33.7 ± 13.3) and healthy control group (mean ± SD: 30 ± 4.52). The proportions of males versus females were, however, not evenly distributed among the groups (*p* = 0.002). While we made efforts to balance the proportions of males and females in healthy and TB groups, challenges were encountered while recruiting females in the HIV mono-infected and HIV-TB co-infected groups. This was primarily because the women in general avoided participating in the study due to social stigma associated with HIV and HIV-TB co-infection.

### 2.2. Ethical Statement

Written informed consent was obtained from all the recruited human subjects and the study was approved by the Institutional Ethics Committee (IEC) of the Post Graduate Institute of Medical Education & Research (PGIMER), Chandigarh, India vide reference number—INT/IEC/2021/SPL-1248.

### 2.3. CD4^+^ T Cell Count and Plasma RNA Viral Load Estimation

The absolute counts of CD4^+^ T lymphocytes were measured in the erythrocyte-lysed peripheral blood of individuals from HIV-1 subtype-C mono-infected as well as HIV-TB co-infected groups, by flow cytometry using BD Tritest^TM^ CD3^+^ FITC/CD4^+^ PE/CD45^+^ PerCP reagent and BD Trucount^TM^ tubes (BD Bioscience, San Jose, CA, USA). The HIV-1 plasma viral load was quantified in these individuals using the COBAS Taqman HIV-1 test (Roche, Branchburg, NJ, USA) as per the manufacturer’s guidelines.

### 2.4. PBMC Isolation and Semi-Quantitative Expression of Immune Checkpoint Molecules (ICMs) by Real-Time PCR

For the isolation of peripheral blood mononuclear cells (PBMCs) by density gradient centrifugation, the EDTA anti-coagulated venous blood was diluted in plain Roswell Park Memorial Institute (RPMI) medium (Sigma-Aldrich, Ref. 6504-10L, St. Louis, MO, USA) at a ratio of 1:1 and the mixture was layered onto ficoll (HiSep^TM^ LSM 1073; HiMedia, Ref. LS002-500ML, Nashik, Maharashtra, India) at a ratio of 1:2 (HiSep^TM^ LSM 1073: diluted blood). The contents were centrifuged at 450× *g* for 30 min with the centrifuge machine’s breaks in the ‘off’ mode. The white-colored ring or buffy coat (PBMC-rich), formed at the interphase of plasma and ficoll, was aspirated into a centrifuge tube containing plain RPMI and the cells were centrifuged again at 250× *g* for 10 min. The pellet of cells obtained was resuspended and mixed gently in plain RPMI and centrifuged once again at 150× *g* for 5 min. The cells in the pellet were re-suspended in 1 mL of complete RPMI [containing 10% FBS (fetal bovine serum—GIBCO, Grand Island, NY, USA) and antimycotic and antibacterial antibiotics (Sigma-Aldrich, Ref. P4333-100ML, St. Louis, MO, USA)] and counted in a Neubauer chamber at 1:100 dilution. The viability of PBMCs was assessed under microscopy using trypan blue staining and proceeded for staining with fluorochrome conjugated monoclonal antibodies only when the cell viability was >95%.

For semi-quantitative expression of ICMs by Real-time PCR (qRT-PCR), cells were lysed using TRI Reagent^®^ solution (Sigma-Aldrich, Ref. T9424-200ML, St. Louis, MO, USA), and total RNA was isolated using the chloroform-isopropanol method [58]. Complementary DNA (cDNA) was then synthesized from 1000 ng of total RNA using the RevertAid First Strand cDNA Synthesis Kit (Catalogue no. K1622, Thermo Scientific, Waltham, MA, USA). Real-time PCR was performed in a real-time PCR machine (LightCycler, Roche Diagnostics, Indianapolis, IN, USA) using SYBR Green (Catalogue no. A6001, Promega, Madison, WI, USA). The qRT-PCR protocol included an initial denaturation step at 95 °C for 5 min, followed by 35 cycles of 95 °C for 10 s, 60 °C for 30 s and extension at 72 °C for 15 s. Normalization and fold changes were calculated using the 2^−∆∆Ct^ method and the data analyzed by comparing the Ct values after normalization with the housekeeping gene (β-actin). The gene-specific primers used are listed in Supplementary Table S1.

### 2.5. Frequency of CD4^+^ and CD8^+^ T Cells Surface-Expressing ICMs Using Multiparametric Flow Cytometry

Flow cytometry was used to assess the surface expression of ICMs on CD4^+^ and CD8^+^ T Cells using fluorochrome-conjugated monoclonal antibodies. The following antibodies were procured from BD Biosciences, Franklin Lakes, NJ, USA: anti-CD3 FITC UCHT1 (Cat no. 555916, Lot no. 6285760), anti-CD4 Horizon V500 RPA-T4 (Cat. No. 560768, Lot no. 4053804), anti-CD8 PE Cy7 HIT8A (Cat. No. 566858, Lot no. 1215646), anti-TIM-3 (CD366) PECF594 7D3 (Cat. No. 565560, Lot no. 1214300), anti-TIGIT BV421 741182 (Cat. No. 747844, Lot no. 3186985), anti-PD1 (CD279) APC MIH4 (Cat. No. 558694, Lot no. 1117202), anti-LAG3 (CD223) PE T47-530 (Cat. No. 565616, Lot no. 1033009), and anti-CTLA4 (CD152) BB700 BNI3 (Cat. No. 566901, Lot no. 2005272).

For staining, PBMCs from the recruited human subjects were suspended in staining buffer containing phosphate-buffered saline (PBS) mixed with 1% FBS along with antibody cocktail incubated for 30 min at room temperature in the dark. The antibodies were used at the concentrations recommended by the manufacturer ensuring optimal staining. The cells were washed with PBS containing 1% FBS, centrifuged at 150× *g* for 5 min and resuspended in PBS prior to acquisition. Singlets were selected based on forward scatter area (FSC-A) versus forward scatter height (FSC-H) and lymphocytes were gated based on an FSC-A versus side scatter area (SSC-A) plot to exclude any debris and/or dead cells. Cells were acquired on FACS Aria II flow cytometer (Becton Dickinson, San Jose, CA, USA), compensated using single-stained controls and gated for CD3^+^ CD4^+^ T cells, and CD3^+^ CD8^+^ T cells, expressing any or a combination of TIM-3, LAG-3, PD-1, CTLA-4, and TIGIT (Supplementary Figure S1). The frequency of cells expressing ICMs individually or in combination was assessed on these populations using data analysis software BD FACSDiva^TM^ (version 8.0.2, Franklin Lakes, NJ, USA).

### 2.6. Statistical Analysis

Statistical analysis was conducted using GraphPad Prism software (version 8.0.2, GraphPad Software Inc., La Jolla, CA, USA). Data were visually inspected by means of histograms to detect any outliers. Data were presented as mean ± standard deviation (mean ± SD). The Mann–Whitney U test was used for comparison between two groups and Kruskal–Wallis (KW) test was used for comparisons between multiple groups, followed by Dunn’s multiple comparison adjustment for pairwise comparisons. Correlation coefficients were determined using the Spearman rank sum test. A *p*-value of less than 0.05 was considered statistically significant.

## 3. Results

### 3.1. Comparative Evaluation of Absolute CD4^+^ T Cell Counts and Plasma Viral Load in HIV-Mono- and HIV-TB Co-Infected Individuals

**Figure 1:**
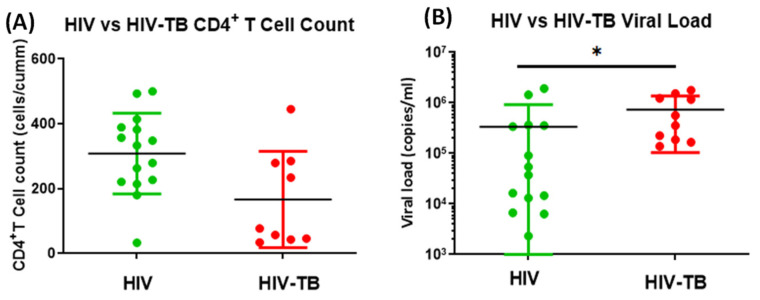
Comparison between the absolute CD4^+^ T cell count and HIV-1 plasma viral load in HIV mono-infected and HIV-TB co-infected individuals. A scatter plot showing comparative absolute CD4^+^ T-cell counts (cells/µL) (**A**) and viral loads (copies/mL) (mean ± SD) (**B**) in HIV (green dots, HIV^+^ mono-infected patients *n* = 15) and HIV-TB (red dots, HIV^+^ Mtb^+^ co-infected patients *n* = 10). Each dot represents one individual. Statistical analysis was performed using Mann–Whitney test. Significance of the data was calculated and denoted as * *p* < 0.05.

At the time of recruitment, the mean absolute CD4^+^ T-cell counts in the peripheral blood (cells/µL) of HIV-TB co-infected individuals were lower (mean ± SD: 166.7 ± 148.4 cells/µL) than that of HIV mono-infected individuals (mean ± SD: 308.9 ± 124.4 cells/µL), although the difference was statistically not significant (Figure 1A). The HIV-1 plasma viral load (copies/mL) was significantly higher in HIV-TB co-infected individuals (mean ± SD: 736,364 ± 632,465 copies/mL) as compared to HIV mono-infected individuals (mean ± SD: 334,212 ± 597,672 copies/mL) (*p *= 0.0229/*; Figure 1B).

### 3.2. Increased Gene Expression of Exhaustion Markers in HIV-TB Co-Infected Individuals as Compared to HIV Mono-Infected Individuals and TB Mono-Infected Individuals

**Figure 2:**
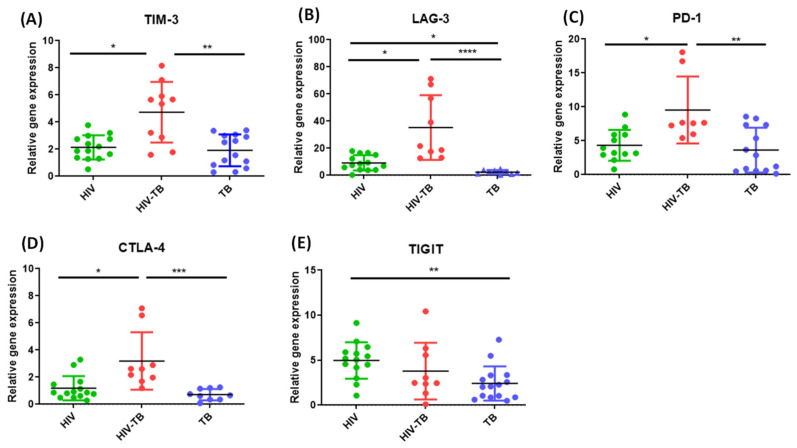
Gene expression profiling of TIM-3, LAG-3, PD-1, CTLA-4 and TIGIT in the peripheral blood of the recruited subjects. The mRNA expression levels of TIM-3 (**A**), LAG-3 (**B**), PD-1 (**C**), CTLA-4 (**D**), and TIGIT (**E**) were measured in PBMCs of HIV (green dots, HIV^+^ mono-infected patients *n* = 15), HIV-TB (red dots, HIV^+^ Mtb^+^ co-infected patients *n* = 10), and TB (blue dots, Mtb^+^ mono-infected patients *n* = 15) relative to controls (healthy controls *n* = 15) by real-time PCR using SYBR Green chemistry. Healthy controls served as baseline reference (calibrator group with fold change = 1) during ∆∆Ct analysis. Each dot represents one individual. Kruskal–Wallis (KW) test was performed for comparisons between multiple groups with pairwise comparisons using Dunn’s multiple comparison adjustment for an overall *p*-values < 0.05. Error bars represent mean ± SD. Level of significance, * *p* < 0.05, ** *p* < 0.01, *** *p* < 0.001 and **** *p* < 0.0001.

In order to gain insights into the overall exhausted state in HIV-TB co-infected individuals, we evaluated the differential gene expression in terms of mRNA abundance of TIM-3, LAG-3, PD-1, CTLA-4 and TIGIT in PBMCs of recruited subjects by semi-quantitative real-time PCR assay after normalizing with housekeeping gene, β-actin. The gene expression (mean ± SD) of TIM-3 (4.73 ± 2.24), LAG-3 (35.3 ± 23.9), PD-1 (9.55 ± 4.93), and CTLA-4 (3.20 ± 2.12) was significantly (*p *= 0.0058, *p *< 0.0001, *p *= 0.0090, *p *= 0.0008, respectively) higher in HIV-TB co-infected individuals as compared to HIV mono-infected individuals (mean ± SD: 2.14 ± 0.89, 9.24 ± 5.62, 4.33 ± 2.28, 1.19 ± 0.894, respectively) as well as TB mono-infected individuals (mean ± SD: 1.91 ± 1.18, 2.46 ± 1.43, 3.64 ± 3.31, 0.71 ± 0.417, respectively) (Figure 2A–D, respectively). The expression (mean ± SD) of LAG-3 (9.24 ± 5.62; *p *< 0.05) and TIGIT (4.98 ± 2.02; *p *= 0.0116) was significantly higher in HIV mono-infected individuals as compared to TB mono-infected individuals (mean ± SD: 3.64 ± 3.31, 2.42 ± 1.90, respectively) (Figure 2B,E, respectively).

**Figure 3:**
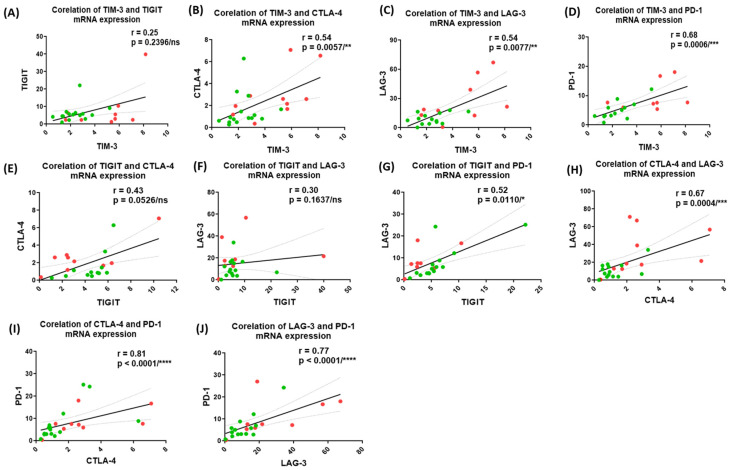
Correlation between the gene (mRNA) expressions of ICMs in HIV mono-infected and HIV-TB co-infected individuals. Spearman’s r correlation between the gene expression of TIM-3 vs. TIGIT (**A**), TIM-3 vs. CTLA-4 (**B**), TIM-3 vs. LAG-3 (**C**), TIM-3 vs. PD-1 (**D**), TIGIT vs. CTLA-4 (**E**), TIGIT vs. LAG-3 (**F**), TIGIT vs. PD-1 (**G**), CTLA-4 vs. LAG-3 (**H**), CTLA-4 vs. PD-1 (**I**), LAG-3 vs. PD-1 (**J**) in HIV-TB co-infected individuals (*n* = 15) and HIV mono-infected individuals (*n* = 10). Red dots represent HIV-TB co-infected individuals and green dots represent HIV mono-infected individuals. Each dot represents one individual. The solid line represents the linear regression of points and dotted lines represent 95% confidence band for their mean values. r, Spearman’s correlation coefficient. Level of significance, ns *p* ≥ 0.05, * *p* < 0.05, ** *p* < 0.01, *** *p* < 0.001 and **** *p* < 0.0001.

Additionally, we also investigated whether there is any correlation among the gene expression levels of ICMs. In HIV-TB co-infected and HIV mono-infected individuals, the expression levels of TIM-3 showed a significant positive Spearman’s r correlation with CTLA-4 (r = 0.54, *p *= 0.0057), LAG-3 (r = 0.54, *p *= 0.0077) and PD-1 (r = 0.68, *p *= 0.0006) (Figure 3B–D, respectively), but not significant with TIGIT (Figure 3A, respectively). Similarly, expression of TIGIT showed significant correlation with PD-1 (r = 0.52, *p *= 0.0110) (Figure 3G), while expression of CTLA-4 showed significant correlation with LAG-3 (r = 0.67, *p *= 0.0004), and PD-1 (r = 0.81, *p* < 0.0001) (Figure 3H,I, respectively) and expression of LAG-3 showed significant correlation with PD-1 (r = 0.77, *p* < 0.0001) (Figure 3J). However, no correlation was observed between expression of TIGIT and CTLA-4, and TIGIT and LAG-3 (Figure 3E,F, respectively).

### 3.3. Increased Frequency of CD4^+^ and CD8^+^ T Cells Surface-Expressing ICMs in HIV-TB Co-Infected Individuals as Compared to Other Groups

To investigate the surface expression of ICMs on immune cells, we used flow cytometry to measure the frequencies of CD3^+^ CD4^+^ and CD3^+^ CD8^+^ T-cell subsets expressing TIM-3, TIGIT, CTLA-4, LAG-3 and PD-1 in the PBMCs isolated from HIV (HIV^+^ mono-infected patients *n* = 15), HIV-TB (HIV-Mtb co-infected patients *n* = 10), TB (Mtb ^+^ mono-infected patients *n* = 15), and Control (healthy volunteers *n* = 15).

**Figure 4:**
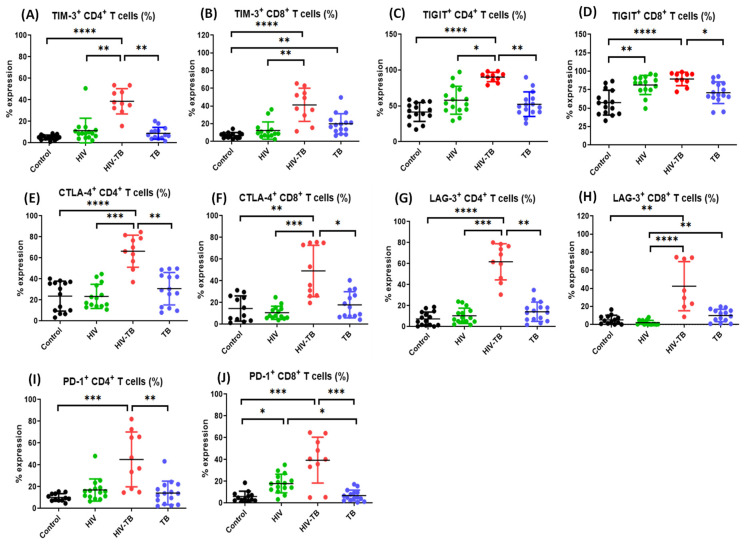
Comparison between the frequencies of ICMs expressing CD4^+^ and CD8^+^ T-cell subsets. The comparative frequencies (%) of CD4^+^ & CD8^+^ T-lymphocytes expressing TIM-3 (**A**,**B**), TIGIT (**C**,**D**), CTLA-4 (**E**,**F**), LAG-3 (**G**,**H**), PD-1 (**I**,**J**), respectively, in Control (black dots, healthy controls *n* = 15), HIV (green dots, HIV^+^ mono-infected patients *n* = 15), HIV-TB (red dots, HIV^+^ Mtb^+^ co-infected patients *n* = 10) and TB (blue dots, Mtb^+^ mono-infected patients *n* = 15) groups. Each dot represents one individual. Statistical analysis was performed using Kruskal–Wallis (KW) test for comparisons between multiple groups with pairwise comparisons using Dunn’s multiple comparison adjustment for an overall *p*-values < 0.05. Data are represented as mean ± SD. Level of significance, * *p* < 0.05, ** *p* < 0.01, *** *p* < 0.001 and **** *p* < 0.0001.

Among HIV-TB co-infected individuals, we observed significantly higher frequencies of TIM-3, CTLA-4 and LAG-3 expressing CD4^+^ and CD8^+^ T cell (Figure 4A,B,E–H, respectively) and TIGIT expressing CD4^+^ T cell (Figure 4C) subsets as compared to HIV mono-infected individuals (*p *< 0.05). However, when compared with healthy control group, the expression of all the five ICMs was found to be significantly upregulated in HIV-TB co-infected individuals (Figure 4A–J, respectively). Additionally, the frequencies of TIM-3 and LAG-3 expressing CD4^+^ T-cells (Figure 4A,G) and TIGIT, CTLA-4 and PD-1 expressing CD4^+^ and CD8^+^ T cells (Figure 4C–F,I,J, respectively) were significantly higher in HIV-TB co-infected individuals as compared to Mtb mono-infected ones (*p *< 0.05).

Among HIV mono-infected individuals, the frequencies of TIGIT and PD-1 expressing CD8^+^ T cells (Figure 4D,J, respectively) were significantly higher as compared to healthy control group. Additionally, the frequency of LAG-3 and PD-1 expressing CD8^+^ T cells (Figure 4H,J, respectively) was higher in HIV mono-infected individuals as compared to TB group and the frequency of TIM-3 expressing CD8^+^ T cells was higher in Mtb mono-infected individuals as compared to control group (Figure 4B).

### 3.4. The Frequencies of ICM-Expressing CD4^+^ and CD8^+^ T Cells Correlate Positively with Plasma Viral Load and Negatively with Absolute CD4^+^ T-Cell Counts in HIV-1-Infected Individuals

We next determined the correlations between the frequencies of CD4^+^ and CD8^+^ T cells expressing ICMs and the HIV disease progression parameters, the absolute CD4^+^ T cell counts and plasma viral load.

**Figure 5:**
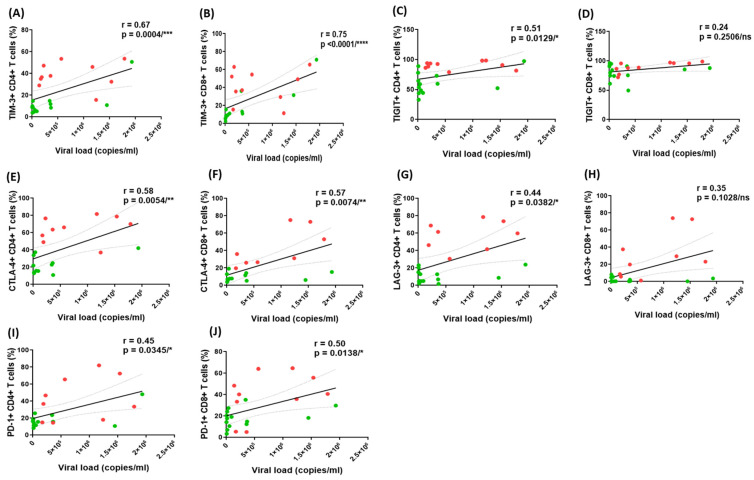
Correlation between the frequencies of CD4^+^ and CD8^+^ T cells expressing ICMs with HIV-1 plasma viral load. Correlations between the frequencies of TIM-3^+^ CD4^+^ (**A**), TIM-3^+^ CD8^+^ (**B**), TIGIT^+^ CD4^+^ (**C**), TIGIT^+^ CD8^+^ (**D**), CTLA-4^+^ CD4^+^ (**E**), CTLA-4^+^ CD8^+^ (**F**), LAG-3^+^ CD4^+^ (**G**), LAG-3^+^ CD8^+^ (**H**), and PD-1^+^ CD4^+^ (**I**), PD-1^+^ CD8^+^ (**J**) and the plasma viral load (VL) in HIV mono-infected and HIV-TB co-infected individuals. Each dot represents one individual. Red dots represent HIV-TB co-infected individuals and green dots represent HIV mono-infected individuals. The solid line represents the linear regression of points and dotted lines represent 95% confidence band for their mean values. r, Spearman’s correlation coefficient. Level of significance, ns *p* ≥ 0.05, * *p* < 0.05, ** *p* < 0.01, *** *p* < 0.001 and **** *p* < 0.0001.

The frequencies of TIM-3^+^ CD4^+^ T cells (r = 0.67, *p *= 0.0004), TIM-3^+^ CD8^+^ T cells (r = 0.75, *p *< 0.0001), TIGIT^+^ CD4^+^T cells (r = 0.51, *p *= 0.0129), CTLA-4 CD4^+^ T cells (r = 0.58, *p *= 0.0054), CTLA-4^+^ CD8^+^ T cells (r = 0.57, *p *= 0.0074), LAG-3^+^ CD4^+^T cells (r = 0.44, *p *= 0.0382), PD-1^+^ CD4^+^ T cells (r = 0.45, *p *= 0.0345) and PD-1^+^ CD8^+^ T cells (r = 0.50, *p *= 0.0138) among all HIV-infected individuals in both groups were positively correlated with plasma viral load (Figure 5A–C,E–G,I, respectively). No significant correlation was observed between the frequencies of TIGIT^+^ CD8^+^ T cells (r = 0.24, *p *= 0.2506) or LAG-3^+^ CD8^+^ T cells (r = 0.35; *p *= 0.1028) with the plasma viral load (Figure 5D,H).

**Figure 6:**
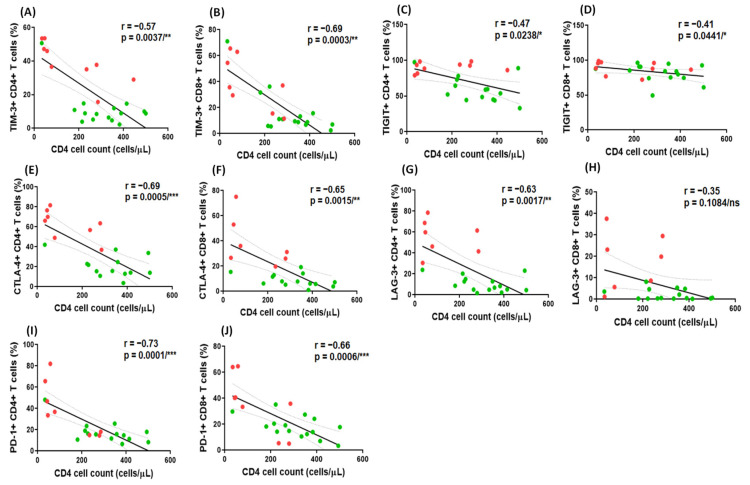
Correlation between the frequencies of CD4^+^ and CD8^+^ T cells expressing ICMs and the absolute CD4^+^ T cell count in peripheral blood. Correlations between the frequencies of TIM-3^+^ CD4^+^ (**A**), TIM-3^+^ CD8^+^ (**B**), TIGIT^+^ CD4^+^ (**C**), TIGIT^+^ CD8^+^ (**D**); CTLA-4^+^ CD4^+^ (**E**), CTLA-4^+^ CD8^+^ (**F**), LAG-3^+^ CD4^+^ (**G**), LAG-3^+^ CD8^+^ (**H**), and PD-1^+^ CD4^+^ (**I**), PD-1^+^ CD8^+^ (**J**) and CD4^+^ T cell count (cells/µL) in HIV mono-infected and HIV-TB co-infected individuals. Red dots represent HIV-TB co-infected individuals and green dots represent HIV mono-infected individuals. Each dot represents one individual. The solid line represents the linear regression of points and dotted lines represent 95% confidence band for their mean values. r, Spearman’s correlation coefficient. Level of significance, ns *p* ≥ 0.05, * *p* < 0.05, ** *p* < 0.01, and *** *p* < 0.001.

The frequencies of TIM-3^+^ CD4^+^ (r = −0.57, *p *= 0.0037), TIM-3^+^ CD8^+^ T cells (r = −0.69, *p *= 0.0003), TIGIT^+^ CD4^+^T cells (r = −0.47, *p *= 0.0238), TIGIT^+^ CD8^+^T cells (r = −0.41, *p *= 0.0441), CTLA-4^+^ CD4^+^ T cells (r = −0.69, *p *= 0.0005), CTLA-4^+^ CD8^+^ T cells (r = −0.65, *p *= 0.0015), LAG-3^+^ CD4^+^ T cells (r = −0.63, *p *= 0.0017), and PD-1^+^ CD4^+^ T cells (r = −0.73, *p *= 0.0001) and PD-1^+^ CD8^+^ T cells (r = −0.66, *p *= 0.0006) in HIV mono-infected and HIV-TB co-infected individuals were negatively correlated with their peripheral blood absolute CD4^+^ T cell count (Figure 6A–G,I,J, respectively). However, no correlation was observed between the frequencies of LAG-3^+^ CD8^+^T cells (r = −0.35, *p *= 0.1084) and the CD4^+^ T cell count (Figure 6H).

### 3.5. Increased Frequencies of CD4^+^ and CD8^+^ T Cells Co-Expressing Two ICMs in HIV-TB Co-Infected Individuals as Compared to HIV Mono-Infected Individuals

To investigate the co-expression of two ICMs on immune cells, we evaluated the frequencies of CD4^+^ and CD8^+^ T cells co-expressing TIM-3 and TIGIT, TIM-3 and PD-1, TIM-3 and LAG-3, TIM-3 and CTLA-4, TIGIT and PD-1, TIGIT and LAG-3, TIGIT and CTLA-4, PD-1 and LAG-3, PD-1 and CTLA-4 and LAG-3 and CTLA-4 by flow cytometry among all the four groups.

**Figure 7:**
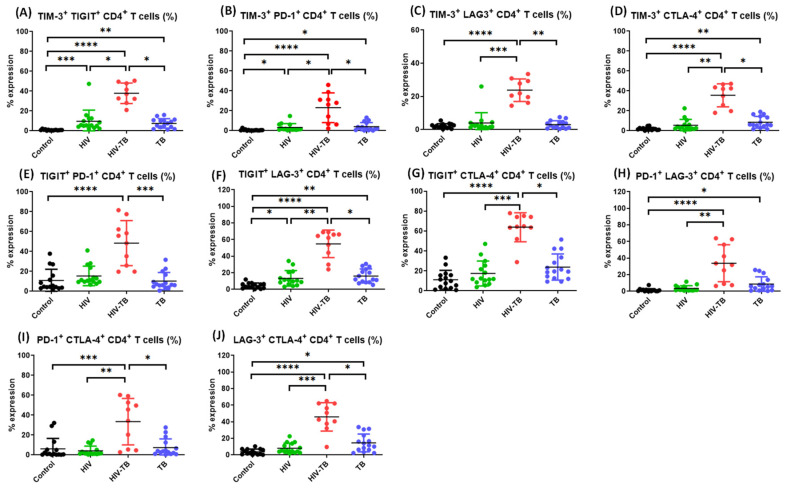
Comparison between the frequencies of CD4^+^ T cells co-expressing dual ICMs in the four groups. Comparison of the frequencies of TIM-3^+^ TIGIT^+^ CD4^+^ T cells (**A**), TIM-3^+^ PD-1^+^ CD4^+^ T cells (**B**), TIM-3^+^ LAG-3^+^ CD4^+^ T cells (**C**), TIM-3^+^ CTLA-4^+^ CD4^+^ T cells (**D**), TIGIT^+^ PD-1^+^ CD4^+^ T cells (**E**), TIGIT^+^ LAG-3^+^ CD4^+^ T cells (**F**), TIGIT^+^ CTLA-4^+^ CD4^+^ T cells (**G**), PD-1^+^ LAG-3^+^ CD4^+^ T cells (**H**), PD-1^+^ CTLA-4^+^ CD4^+^ T cells (**I**) and LAG-3^+^ CTLA-4^+^ CD4^+^ T cells (**J**) among Control (black dots, healthy controls *n* = 15), HIV (green dots, HIV^+^ mono-infected patients *n* = 15), HIV-TB (red dots, HIV^+^ TB^+^ co-infected patients *n* = 10) and TB (blue dots, TB^+^ mono-infected patients *n* = 15). Each dot represents one individual. Statistical analysis was performed using Kruskal–Wallis (KW) test for comparisons between multiple groups with pairwise comparisons using Dunn’s multiple comparison adjustment for an overall *p*-values < 0.05. Data are represented as mean ± SD. Level of significance, * *p* < 0.05, ** *p* < 0.01, *** *p* < 0.001 and **** *p* < 0.0001.

**Figure 8:**
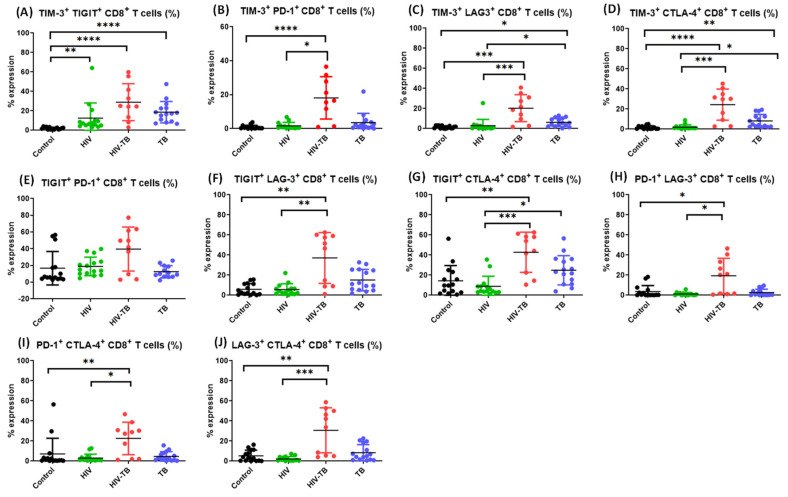
Comparison between the frequencies of CD8^+^ T cells co-expressing dual ICMs in the four groups. Comparison of the frequencies of TIM-3^+^ TIGIT^+^ CD8^+^ T cells (**A**), TIM-3^+^ PD-1^+^ CD8^+^ T cells (**B**), TIM-3^+^ LAG-3^+^ CD8^+^ T cells (**C**), TIM-3^+^ CTLA-4^+^ CD8^+^ T cells (**D**), TIGIT^+^ PD-1^+^ CD8^+^ T cells (**E**), TIGIT^+^ LAG-3^+^ CD8^+^ T cells (**F**), TIGIT^+^ CTLA-4^+^ CD8^+^ T cells (**G**), PD-1^+^ LAG-3^+^ CD8^+^ T cells (**H**), PD-1^+^ CTLA-4^+^ CD8^+^ T cells (**I**) and LAG-3^+^ CTLA-4^+^ CD8^+^ T cells (**J**) among Control (black dots, healthy controls *n* = 15), HIV (green dots, HIV^+^ mono-infected patients *n* = 15), HIV-TB (red dots HIV^+^ TB^+^ co-infected patients *n* = 10) and TB (blue dots, TB^+^ mono-infected patients *n* = 15). Each dot represents one individual. Statistical analysis was performed using Kruskal–Wallis (KW) test for comparisons between multiple groups with pairwise comparisons using Dunn’s multiple comparison adjustment for an overall *p*-values < 0.05. Data are represented as mean ± SD. Level of significance, * *p* <0.05, ** *p* < 0.01, *** *p* < 0.001 and **** *p* < 0.0001.

In HIV-TB co-infected individuals, we observed that the frequencies of CD4^+^ T cells co-expressing TIM-3 and PD-1, TIM-3 and LAG-3, TIM-3 and CTLA-4, TIGIT and LAG-3, TIGIT and CTLA-4, PD-1 and LAG-3, PD-1 and CTLA-4 and LAG-3 and CTLA-4 (Figure 7B–D,F–J, respectively) as well as CD8^+^ T cells (Figure 8B–D,F–J, respectively) and TIM-3^+^ TIGIT^+^ CD4^+^ T cells (Figure 7A) were significantly higher as compared to HIV mono-infected individuals.

Further, the frequencies of CD4^+^ and CD8^+^ T cells co-expressing two ICMs together were also correlated positively with plasma viral load (Supplementary Figure S2A–T) and negatively with absolute CD4^+^ T-Cell counts (Supplementary Figure S3A–T) in HIV mono-infected and HIV-TB co-infected individuals.

## 4. Discussion

T-cell exhaustion is the hallmark of cancers as well as many chronic infections. With the increased recognition of the significance of targeting ICMs for restoration of T-cell effector functions, this strategy offers a promising therapeutic approach. In recent years, various studies have reported that during chronic HIV infection, upregulated expression of ICMs leads to functional exhaustion of CD8^+^ T-cells and loss of effector functions [27,59,60,61]. Based on these observations, several clinical trials are currently being conducted focusing on the safe usage of immune-checkpoint inhibitors (ICIs) as a strategy to enhance HIV-specific immune responses and to target the HIV reservoir in PLHIV [62,63,64,65,66]. These clinical trials made a rationale and prompted us to explore the modulation of the expression of various ICMs on immune cells, possibly mediated by Mtb, in the individuals co-infected with HIV-Mtb, in relation to the rate of HIV disease progression towards AIDS as compared to the individuals mono-infected with HIV-1 only. This could offer key insights into the immunopathogenic mechanisms of HIV disease associated with faster progression to AIDS with increased mortality in HIV-TB co-infected individuals.

Some previous seminal studies on HIV-TB co-infected cohorts from our laboratory as well as others have indicated Mtb-mediated mechanisms causing enhanced propagation and genetic diversity as possible leading causes of increased viral replication and diversity in the lung segments with Mtb infection [67,68,69] and ‘spill-over’ of these genetically altered quasi-species of HIV from the lungs, migrating to systemic circulation, eventually results in viral diversity [69,70]. Similar previous studies from our group have also shown the emergence of genetically altered viral quasi-species, having accumulated significantly higher frequency of high-risk drug resistance (DR) mutations in reverse transcriptase (RT) gene and additional copies of Nuclear Factor kappa-light-chain-enhancer of activated B cells (NF-kB) binding sites in the long terminal repeat (LTR) region of the genome of viral isolates from HIV-TB co-infected individuals, as compared to viral isolates from HIV mono-infected host [71,72]. It has also been reported that Mtb-infected alveolar macrophages produce high levels of inflammatory cytokines like TNF-α, IL-1β, and IL-6, which enhance HIV replication and its persistence in macrophages [4,73,74]. And also, the elevated levels of indoleamine 2,3, dioxygenase (IDO) along with reduced tryptophan in the macrophages of HIV-infected individuals co-infected with Mtb correlate with poor outcomes [75], possibly because of suppressed T-cell responses along with higher IL-10 and low IL-2 production, which further impairs the immune system [4]. These changes in the emerging viral species in co-infected individuals collectively may be contributing to enhanced viral replication and increased cell death in co-infected individuals, yet the role of Mtb in host immune responses in these individuals has not been clearly understood. In the present study, we have attempted to further understand the impact of Mtb on modulation of host factors, specifically the expression of ICMs on immune cells in relation to increased survival and replication of virus in the co-infected host in comparison to HIV mono-infected host. The study has been conducted in cohorts of HIV-infected human subjects, either alone or co-infected with Mtb from North India, where HIV-1 subtype C has been found to be predominant [71,72,76,77]. The study population is unique in the sense that no serious attempts have been made to understand the pathogenesis of HIV disease in this population, living in highly endemic geographical region for tuberculosis, and having a very high burden of HIV-TB co-infection. Most of the studies reported previously in literature have been conducted in other parts of world, where HIV Subtype-B is more prevalent, and incidence of TB is also low [29,40,51,52,54,55,56]. The findings of index study would be important with significantly high translational value, especially for countries which are having high burden of these two infections.

Moreover, our study has been conducted in treatment-naïve patients, because the start of ART or ATT would have changed the immune status, eluding fair assessment of Mtb-mediated immune modulation in these subjects. By focusing on untreated individuals, we aimed to capture the true immunopathological profile of HIV-TB co-infection. A cross-sectional assessment of absolute CD4^+^ T-cell count and plasma viral load, in our recruited study subjects, revealed a lower mean absolute CD4^+^ T-cell count and significantly higher mean plasma viral load in HIV-TB co-infected individuals in comparison to HIV mono-infected individuals, confirming the disease severity and propensity of a faster HIV disease progression in co-infected individuals when compared to HIV-only infected ones, in these study groups. These results align with a previous report that shows the Mtb co-infection leads to higher immune activation, higher viral set point threshold, higher levels of viral RNA and cells harboring integrated viral DNA, thus promoting faster HIV disease progression in co-infected individuals [68].

The gene expression of ICMs, TIM-3, LAG-3, PD-1 and CTLA-4 in terms of mRNA abundance was found to be significantly upregulated in PBMCs of HIV-TB co-infected individuals as compared to HIV mono-infected and TB mono-infected individuals in our study subjects, indicating a dysregulated expression of ICMs in co-infected host. Interestingly, a positive correlation among the gene expression of ICMs, as assessed by Spearman’s r correlation analysis, strongly suggests that expression of ICMs is co-regulated as part of immune regulatory network. This observation falls in line with findings from previous studies that mRNA expression levels of TIM-3 correlate positively with PD-1 and LAG-3 [78] and that gene expression of PD-1, LAG-3, TIM-3 and TIGIT form a transcriptionally co-regulated module in T cells [79]. The possible mechanisms for sustained and coregulated expression of ICMs may include shared signaling pathways and transcription factors along with chronic antigen exposure in HIV-TB co-infection leading to a state of immune exhaustion, where multiple ICMs are up-regulated as a part of the exhaustion phenotype to prevent further immune activation. Furthermore, evidence from recent studies suggests that impaired TNF-α signaling in alveolar macrophages, predominately M2-like alveolar macrophages and aberrant macrophage-T cell crosstalk, may impair effective immune responses and microbial clearance [80] and can also impact downstream pathways involving NF-kB and other transcription factors known to regulate ICMs expression. This dysfunction likely promotes chronic inflammation and immune dysregulation, thereby contributing to sustained ICM expression.

While gene expression gives crucial information related to regulatory mechanisms, it is the surface expression of ICMs on immune cells that causes the functional suppression of T-cell activation and downregulated effector mechanisms. In order to assess the impact of Mtb on immune cell functions, we subsequently assessed the surface expression of ICMs on immune cells by flow cytometry and observed that the frequencies of both CD4^+^ as well as CD8^+^ T cells expressing ICMs like TIM-3, CTLA-4 and LAG-3 and CD4^+^ T cells expressing TIGIT were significantly increased in HIV-TB co-infected individuals as compared to HIV mono-infected individuals. Furthermore, the expression of all the checkpoint molecules was found to be significantly higher across both CD4^+^ and CD8^+^ T cells in HIV-TB co-infected individuals as compared to healthy controls, while the frequencies of both CD4^+^ as well as CD8^+^ T cells expressing ICMs like TIGIT, CTLA-4 and PD-1 and CD4^+^ T cells expressing TIM-3 and LAG-3 were significantly increased in HIV-TB co-infected individuals as compared to TB mono-infected individuals, indicating an enhanced level of immune-exhaustion in HIV-TB co-infected hosts as compared to HIV or Mtb mono-infected ones. These findings are consistent with previous reports indicating total T-lymphocytes expressing PD-1 were upregulated in both HIV and HIV-TB co-infected individuals as compared to TB mono-infected individuals and healthy controls [51]. These findings suggest that the substantial differences in the ICM expression profiles among HIV mono-infected and HIV-TB co-infected individuals may be attributed to the enhanced inflammatory microenvironment induced by Mtb co-infection, which eventually modulates the expression of these molecules [81]. Consistent with previously published studies in HIV mono-infected individuals [27,28,32,33,36,82], our results also showed that the frequencies of CD4^+^ as well as CD8^+^ T-cells expressing various ICMs showed a significant positive correlation with plasma viral load and negative correlation with absolute CD4^+^ T cell count, both of which are key parameters of HIV disease progression. This suggests that the observed upregulated expression of ICMs in HIV-TB co-infected individuals might be the major factor contributing to the accelerated disease progression [27,28,32,33]. The results are also in agreement with other previously published studies, which have also indicated that frequency of PD-1^+^ CD4^+^ T-cell subset in HIV-infected individuals is inversely correlated with absolute CD4^+^ T-cell counts and positively with HIV-1 viral load [27] and proportions of CTLA-4 expressing CD4^+^ T cells were inversely correlated to CD4^+^ T cell counts [28]. These results, along with findings from our study, thus strongly suggest the therapeutic potential of targeting these upregulated ICMs to restore the T cell functions and improve disease outcomes in pursuit of better management of HIV-TB co-infected individuals.

Although most chronic infections and cancers are associated with global immune suppression, immune cells, especially T cells, are not necessarily physically lost [83] but instead enter non-functional, poorly functional, or exhausted states [84,85]. The extent of CD8^+^ T cell exhaustion is determined by the consistently high levels of viral antigens and availability of CD4^+^ T cells. The CD4^+^ T cells are also known to succumb to exhaustion in such chronic viral infections, which can further weaken the anti-viral CD8^+^ T cell responses. The CD8^+^ T cell exhaustion is often characterized by the gradual and progressive loss of effector capabilities, the sustained upregulation of multiple inhibitory receptors, and the loss of self-renewal abilities, which compromise the ability to control viral infections [86]. The co-expression of multiple ICMs on CD4^+^ or CD8^+^ T cells has been indicated to give rise to a severely exhausted phenotype. Notably, the TIM-3^+^ PD-1^+^ dual positive tumor-infiltrating T lymphocytes (TILs) are defined by their failure to proliferate with reduced expression of IL-2, TNF-α, and IFN-γ and these cells exhibit the most pronounced exhausted state [87]. Multiple studies indicate that targeted inhibition of both TIM-3 and PD-1 signaling pathways together has been more effective in enhancing T cell responses and viral suppression in patients with various chronic infections [87,88,89]. Several pre-clinical studies in both cancer research [17,87,90] as well as in HIV infections [64,91] have also supported the effectiveness of targeting two ICMs simultaneously as compared with targeting either one alone.

It was very interesting to find that the frequencies of both CD4^+^ as well as CD8^+^ T cells co-expressing two ICMs together was significantly higher in HIV-TB co-infected individuals in our study, as compared to the HIV mono-infected group, which indicates that Mtb further modulates the upregulated expression of multiple co-inhibitory molecules and pushes the co-infected host further into a deeply immune-compromised state, making a favorable microenvironment for the survival and proliferation of both bugs together. This was further substantiated by the observation of a significant positive correlation between the frequencies of CD4^+^ and CD8^+^ T cells co-expressing two ICMs and the HIV plasma viral load along, with a negative correlation with the absolute CD4^+^ T-cell count. All together, these results indicate a direct relationship of the expanded population of terminally exhausted immune cells having increased expression of multiple ICMs, associated with faster HIV disease progression in HIV-TB co-infected individuals. Our observations corroborate the findings of a previous study that reported dual-positive PD-1^+^CD39^+^CD8^+^ T cells showing negative correlation with CD4^+^ T-cell counts, and positive correlation with viral load [91].

The study thus highlights the possible therapeutic potential of targeting these upregulated ICMs to restore T cell functions and improve disease outcomes for better management of HIV-TB co-infected individuals. One limitation of this study remains the small number of study subjects from a single center in India. That vouches for a study on a larger population in a multi-centric approach, focusing on comprehensive analysis of immune status of patients, including characterization of various other cellular subsets, to substantiate these findings. Moreover, future studies from our group aim to delve deeper into the molecular mechanisms modulated by Mtb, causing upregulated expression of ICMs in HIV-TB co-infection.

## 5. Conclusions

In conclusion, this study provides insight into potentially targetable chronic immunosuppressive mechanisms in HIV-TB co-infection. Our findings highlight the concerning persistence of elevated immune dysfunction in co-infected individuals. Blocking of ICMs using a combination of checkpoint inhibitors and/or small molecule inhibitors targeting ICMs may restore the functionality of immune cells and enhance the ability of the immune system to control the virus, while improving the clearance of both HIV and Mtb bugs. These insights may have far-reaching clinical implications in devising better management strategies to alleviate the adversities of Mtb co-infection among HIV-infected individuals, while also suggesting novel therapeutic approaches that could further be explored. The study findings indicate that this strategy could be used as an adjunct therapy to further improve the efficacy of conventional ART and ATT in co-infected individuals, at the same time emphasizing on the importance of conducting similar research across diverse geographic regions.

## Figures and Tables

**Table 1:** Characteristics of study participants recruited in four groups.

Characteristics of Study Participants	HIV Mono-Infected	HIV-TB Co-Infected	TB Mono-Infected	Healthy Controls
Human subjects (*n*)	15	10	15	15
Mean age (years)	35.2	39	33.67	30
Males (%)	73.33%	80%	53.33%	60%
Females (%)	26.67%	20%	46.67%	40%
Mean CD4 count (cells/μL)/(Range)	308.9	166.7 *	NA	NA
Mean CD4 T-cell count (Range)	(33–500)	(34–445) *	NA	NA
Median CD4 T-cell count (cells/μL)	333	77 *	NA	NA
Median CD4 T-cell count (cells/μL)/[IQR]	[221–389]	[44.5–282] *	NA	NA
Mean viral load (copies/mL)	334,212	736,364	NA	NA
Mean viral load (copies/mL)/(Range)	(TND−1,930,480)	(138,183–1,787,488)	NA	NA
Median viral load (copies/mL)	45,749	460,125	NA	NA
Median viral load (copies/mL)/[IQR]	[11,543–358,292]	[181,538–1,311,977]	NA	NA

Abbreviations: Interquartile Range (IQR), not available (NA), target not detected (TND). * One participant did not have a pre-treatment CD4 count available.

## Data Availability

The original contributions presented in the study are included in the article/Supplementary Materials. For further inquiries, please contact the corresponding author.

## Supplementary Materials

The following supporting information can be downloaded at: https://www.mdpi.com/article/10.3390/pathogens14080802/s1, Table S1: Primer sequences, amplicon size and respective optimal annealing temperature for each Immune checkpoint molecules (ICMs) and housekeeping genes; Figure S1: Gating strategy used to identify CD4^+^ T helper and CD8^+^ cytotoxic T-lymphocytes expressing ICMs in human peripheral blood; Figure S2: Correlation between the frequencies of CD4^+^ and CD8^+^ T cells co-expressing dual ICMs with the HIV-1 plasma viral load; Figure S3: Correlation between the frequencies of CD4^+^ and CD8^+^ T cells co-expressing dual ICMs with the absolute CD4^+^ T-cell counts.

## Abbreviations

The following abbreviations are used in this manuscript:

AIDS	Acquired immunodeficiency syndrome
ART	antiretroviral therapy
ATT	anti-tuberculosis treatment
CAD	coronary artery disease
CCR5	C-C Motif Chemokine Receptor 5
cDNA	Complementary DNA
CKD	chronic kidney disease
CTLA-4	Cytotoxic T-Lymphocyte Antigen-4
CXCR4	C-X-C Motif Chemokine Receptor 4
FBS	Fetal bovine serum
FSC-A	forward scatter area
FSC-H	forward scatter height
HIV	Human immunodeficiency virus
HO-1	heme-oxygenase-1
ICIs	Immune checkpoint inhibitors
ICMs	immune checkpoint molecules
ICOS	Inducible T-cell Costimulator
ICTC	Integrated Counselling and Testing Centre
IDO	Indoleamine 2,3, dioxygenase
IDUs	intravenous drug users
IEC	Institutional Ethics Committee
IFN-γ	Interferon-gamma
iNKT	Invariant natural killer T cells
IQR	Interquartile Range
KW	Kruskal-Wallis
LAG-3	Lymphocyte Activation Gene-3
LTR	long terminal repeat
Mtb	*Mycobacterium tuberculosis*
NACO	National AIDS Control Organization
NF-kB	Nuclear Factor kappa-light-chain-enhancer of activated B cells
NK cells	Natural killer cells
PBMCs	Peripheral blood mononuclear cells
PBS	Phosphate-Buffered Saline
PD-1	Programmed cell death 1
PLHA	people living with HIV/AIDS
qRT-PCR	Quantitative real-time polymerase chain reaction
RT	reverse transcriptase
SD	Standard deviation
SSC-A	side scatter area
TB-IRIS	TB-associated immune reconstitution inflammatory syndrome
TIGIT	T cell immunoreceptor with Ig and immunoreceptor tyrosine-based inhibitory motif domains
TIM-3	T-cell Immunoglobulin and Mucin-domain containing-3
TND	Target not detected
TNF-α	tumour necrosis factor-alpha
WHO	World Health Organization

## References

[1] https://iris.who.int/bitstream/handle/10665/376584/9789240087002-eng.pdf?sequence=1.

[2] Global Tuberculosis Report 2024. https://www.who.int/teams/global-tuberculosis-programme/tb-reports/global-tuberculosis-report-2024.

[3] https://tbcindia.mohfw.gov.in/wp-content/uploads/2023/05/6250311444TB-India-Report-2018.pdf.

[4] Shankar E.M., Vignesh R., Ellegård R., Barathan M., Chong Y.K., Bador M.K., Rukmani D.V., Saber N.S., Kamarulzaman A., Velu V. (2014). HIV-*Mycobacterium tuberculosis* co-infection: A “danger-couple model” of disease pathogenesis. Pathog. Dis..

[5] Leitner J., Grabmeier-Pfistershammer K., Steinberger P. (2010). Receptors and ligands implicated in human T cell costimulatory processes. Immunol. Lett..

[6] Larsson M., Shankar E.M., Che K.F., Saeidi A., Ellegård R., Barathan M., Velu V., Kamarulzaman A. (2013). Molecular signatures of T-cell inhibition in HIV-1 infection. Retrovirology.

[7] O’Neill R.E., Cao X. (2019). Co-stimulatory and co-inhibitory pathways in cancer immunotherapy. Adv. Cancer Res..

[8] Zhang Q., Vignali D.A.A. (2016). Co-stimulatory and co-inhibitory pathways in autoimmunity. Immunity.

[9] Capece D., Verzella D., Fischietti M., Zazzeroni F., Alesse E. (2012). Targeting Costimulatory Molecules to Improve Antitumor Immunity. BioMed Res. Int..

[10] Schnell A., Bod L., Madi A., Kuchroo V.K. (2020). The yin and yang of co-inhibitory receptors: Toward anti-tumor immunity without autoimmunity. Cell Res..

[11] Kamali A.N., Bautista J.M., Eisenhut M., Hamedifar H. (2023). Immune checkpoints and cancer immunotherapies: Insights into newly potential receptors and ligands. Ther. Adv. Vaccines Immunother..

[12] Guo Z., Zhang R., Yang A.-G., Zheng G. (2023). Diversity of immune checkpoints in cancer immunotherapy. Front. Immunol..

[13] He X., Xu C. (2020). Immune checkpoint signaling and cancer immunotherapy. Cell Res..

[14] Cai H., Liu G., Zhong J., Zheng K., Xiao H., Li C., Song X., Li Y., Xu C., Wu H. (2020). Immune Checkpoints in Viral Infections. Viruses.

[15] Wykes M.N., Lewin S.R. (2018). Immune checkpoint blockade in infectious diseases. Nat. Rev. Immunol..

[16] Cai X., Zhan H., Ye Y., Yang J., Zhang M., Li J., Zhuang Y. (2021). Current Progress and Future Perspectives of Immune Checkpoint in Cancer and Infectious Diseases. Front. Genet..

[17] Turnis M.E., Andrews L.P., Vignali D.A.A. (2015). Inhibitory receptors as targets for cancer immunotherapy. Eur. J. Immunol..

[18] McDermott D.F., Atkins M.B. (2013). PD-1 as a potential target in cancer therapy. Cancer Med..

[19] Iwai Y., Hamanishi J., Chamoto K., Honjo T. (2017). Cancer immunotherapies targeting the PD-1 signaling pathway. J. Biomed. Sci..

[20] Postow M.A., Callahan M.K., Wolchok J.D. (2015). Immune Checkpoint Blockade in Cancer Therapy. J. Clin. Oncol..

[21] Zhang P., Wang Y., Miao Q., Chen Y. (2023). The therapeutic potential of PD-1/PD-L1 pathway on immune-related diseases: Based on the innate and adaptive immune components. Biomed. Pharmacother..

[22] Chen R.-Y., Zhu Y., Shen Y.-Y., Xu Q.-Y., Tang H.-Y., Cui N.-X., Jiang L., Dai X.-M., Chen W.-Q., Lin Q. (2023). The role of PD-1 signaling in health and immune-related diseases. Front. Immunol..

[23] Jubel J.M., Barbati Z.R., Burger C., Wirtz D.C., Schildberg F.A. (2020). The Role of PD-1 in Acute and Chronic Infection. Front. Immunol..

[24] Schönrich G., Raftery M.J. (2019). The PD-1/PD-L1 Axis and Virus Infections: A Delicate Balance. Front. Cell. Infect. Microbiol..

[25] Sachdeva M., Fischl M.A., Pahwa R., Sachdeva N., Pahwa S. (2010). Immune exhaustion occurs concomitantly with immune activation and decrease in regulatory T cells in viremic chronically HIV-1-infected patients. J. Acquir. Immune. Defic. Syndr..

[26] Muthumani K., Choo A.Y., Shedlock D.J., Laddy D.J., Sundaram S.G., Hirao L., Wu L., Thieu K.P., Chung C.W., Lankaraman K.M. (2008). Human Immunodeficiency Virus Type 1 Nef Induces Programmed Death 1 Expression through a p38 Mitogen-Activated Protein Kinase-Dependent Mechanism. J. Virol..

[27] Day C.L., Kaufmann D.E., Kiepiela P., Brown J.A., Moodley E.S., Reddy S., Mackey E.W., Miller J.D., Leslie A.J., DePierres C. (2006). PD-1 expression on HIV-specific T cells is associated with T-cell exhaustion and disease progression. Nature.

[28] Leng Q., Bentwich Z., Magen E., Kalinkovich A., Borkow G. (2002). CTLA-4 upregulation during HIV infection: Association with anergy and possible target for therapeutic intervention. AIDS.

[29] Kaufmann D.E., Kavanagh D.G., Pereyra F., Zaunders J.J., Mackey E.W., Miura T., Palmer S., Brockman M., Rathod A., Piechocka-Trocha A. (2007). Upregulation of CTLA-4 by HIV-specific CD4^+^ T cells correlates with disease progression and defines a reversible immune dysfunction. Nat. Immunol..

[30] Finney C.A.M., Ayi K., Wasmuth J.D., Sheth P.M., Kaul R., Loutfy M., Kain K.C., Serghides L. (2013). HIV Infection Deregulates Tim-3 Expression on Innate Cells: Combination Antiretroviral Therapy Results in Partial Restoration. JAIDS J. Acquir. Immune Defic. Syndr..

[31] de Kivit S., Lempsink L.J.R., Plants J., Martinson J., Keshavarzian A., Landay A.L. (2015). Modulation of TIM-3 expression on NK and T cell subsets in HIV immunological non-responders. Clin. Immunol..

[32] Jones R.B., Ndhlovu L.C., Barbour J.D., Sheth P.M., Jha A.R., Long B.R., Wong J.C., Satkunarajah M., Schwenekar M., Chapman J.M. (2008). Tim-3 expression defines a novel population of dysfunctional T cells with highly elevated frequencies in progressive HIV-1 infection. J. Exp. Med..

[33] Tian X., Zhang A., Qiu C., Wang W., Yang Y., Qiu C., Liu A., Lingyan Z., Songhua Y., Huiliang H. (2015). The upregulation of LAG-3 on T cells defines a subpopulation with functional exhaustion and correlates with disease progression in HIV-infected subjects. J. Immunol..

[34] Juno J.A., Stalker A.T., Waruk J.L., Oyugi J., Kimani M., Plummer F.A., Kimani J., Fowke K.R. (2015). Elevated expression of LAG-3, but not PD-1, is associated with impaired iNKT cytokine production during chronic HIV-1 infection and treatment. Retrovirology.

[35] Vendrame E., Seiler C., Ranganath T., Zhao N.Q., Vergara R., Alary M., Labbé A.C., Guédou F., Poudrier J., Holmes S. (2020). TIGIT is upregulated by HIV-1 infection and marks a highly functional adaptive and mature subset of natural killer cells. AIDS.

[36] Yin X., Liu T., Wang Z., Ma M., Lei J., Zhang Z., Fu S., Fu Y., Hu Q., Ding H. (2018). Expression of the Inhibitory Receptor TIGIT Is Up-Regulated Specifically on NK Cells With CD226 Activating Receptor From HIV-Infected Individuals. Front. Immunol..

[37] Porichis F., Hart M.G., Massa A., Everett H.L., Morou A., Richard J., Brassard N., Veillette M., Hassan M., Ly N.L. (2018). Immune Checkpoint Blockade Restores HIV-Specific CD4 T Cell Help for NK Cells. J. Immunol..

[38] Brunet-Ratnasingham E., Morou A., Dubé M., Niessl J., Baxter A.E., Tastet O., Brassard N., Ortega-Delgado G., Charlebois R., Freeman G.J. (2022). Immune checkpoint expression on HIV-specific CD4^+^ T cells and response to their blockade are dependent on lineage and function. EBioMedicine.

[39] Balasko A.L., Kowatsch M.M., Graydon C., Lajoie J., Fowke K.R. (2023). The effect of blocking immune checkpoints LAG-3 and PD-1 on human invariant Natural Killer T cell function. Sci. Rep..

[40] Van der Sluis R.M., Kumar N.A., Pascoe R.D., Zerbato J.M., Evans V.A., Dantanarayana A.I., Anderson J.L., Sékaly R.P., Fromentin R., Chomont N. (2020). Combination Immune Checkpoint Blockade to Reverse HIV Latency. J. Immunol..

[41] Fromentin R., DaFonseca S., Costiniuk C.T., El-Far M., Procopio F.A., Hecht F.M., Hoh R., Deeks S.G., Hazuda D.J., Lewin S.R. (2019). PD-1 blockade potentiates HIV latency reversal ex vivo in CD4^+^ T cells from ART-suppressed individuals. Nat. Commun..

[42] Day C.L., Abrahams D.A., Bunjun R., Stone L., de Kock M., Walzl G., Wikinson R.J., Burgers W.A., Hanekom W.A. (2018). PD-1 Expression on *Mycobacterium tuberculosis*-Specific CD4 T Cells Is Associated With Bacterial Load in Human Tuberculosis. Front. Immunol..

[43] Pan S.-W., Shu C.-C., Huang J.-R., Lee C.-C., Tseng Y.-H., Hung J.-J., Hsu P.K., Chen N.J., Su W.J., Feng J.Y. (2022). PD-L1 Expression in Monocytes Correlates with Bacterial Burden and Treatment Outcomes in Active Pulmonary Tuberculosis. Int. J. Mol. Sci..

[44] Suarez G.V., Melucci Ganzarain C.D.C., Vecchione M.B., Trifone C.A., Marín Franco J.L., Genoula M., Moraña E.J., Balboa L., Quiroga M.F. (2019). PD-1/PD-L1 Pathway Modulates Macrophage Susceptibility to *Mycobacterium tuberculosis* Specific CD8^+^ T cell Induced Death. Sci. Rep..

[45] McNab F.W., Berry M.P.R., Graham C.M., Bloch S.A.A., Oni T., Wilkinson K.A., Wilkinson R.J., Kon O.M., Banchereau J., Chaussabel D. (2011). Programmed death ligand 1 is over-expressed by neutrophils in the blood of patients with active tuberculosis. Eur. J. Immunol..

[46] Wang F., Hou H., Wu S., Tang Q., Huang M., Yin B., Huang J., Liu W., Mao L., Lu Y. (2015). Tim-3 pathway affects NK cell impairment in patients with active tuberculosis. Cytokine.

[47] Qiu Y., Chen J., Liao H., Zhang Y., Wang H., Li S., Luo Y., Fang D., Li G., Zhou B. (2012). Tim-3-expressing CD4^+^ and CD8^+^ T cells in human tuberculosis (TB) exhibit polarized effector memory phenotypes and stronger anti-TB effector functions. PLoS Pathog..

[48] Phillips B.L., Gautam U.S., Bucsan A.N., Foreman T.W., Golden N.A., Niu T., Kaushal D., Mehra S. (2017). LAG-3 potentiates the survival of *Mycobacterium tuberculosis* in host phagocytes by modulating mitochondrial signaling in an in-vitro granuloma model. PLoS ONE.

[49] Tezera L.B., Bielecka M.K., Ogongo P., Walker N.F., Ellis M., Garay-Baquero D.J., Thomas K., Reichmann M.T., Johnston D.A., Wilkinson K.A. (2020). Anti-PD-1 immunotherapy leads to tuberculosis reactivation via dysregulation of TNF-α. Elife.

[50] Liu C.-W., Wu L.S.-H., Lin C.-J., Wu H.-C., Liu K.-C., Lee S.-W. (2024). Association of tuberculosis risk with genetic polymorphisms of the immune checkpoint genes PDCD1, CTLA-4, and TIM3. PLoS ONE.

[51] Jurado J.O., Pasquinelli V., Alvarez I.B., Martínez G.J., Laufer N., Sued O., Cahn P., Musella R.M., Abbate E., Salomón H. (2012). ICOS, SLAM and PD-1 expression and regulation on T lymphocytes reflect the immune dysregulation in patients with HIV-related illness with pulmonary tuberculosis. J. Int. AIDS Soc..

[52] Barham M.S., Abrahams D.A., Khayumbi J., Ongalo J., Tonui J., Campbell A., de Kock M., Ouma S.G., Odhiambo F.H., Hanekom W.A. (2019). HIV Infection Is Associated With Downregulation of BTLA Expression on *Mycobacterium tuberculosis*-Specific CD4 T Cells in Active Tuberculosis Disease. Front. Immunol..

[53] Ramaseri Sunder S., Suryadevara N.C., Pydi S.S., Neela V.S.K., Valluri V.L. (2020). Defective Antigen Presentation Leads to Upregulation of PD1 and IL-10 in HIV-TB Co-Infection. J. Interferon Cytokine Res..

[54] Pollock K.M., Montamat-Sicotte D.J., Grass L., Cooke G.S., Kapembwa M.S., Kon O.M., Sampson R.D., Taylor G.P., Lalvani A. (2016). PD-1 Expression and Cytokine Secretion Profiles of *Mycobacterium tuberculosis*-Specific CD4^+^ T-Cell Subsets; Potential Correlates of Containment in HIV-TB Co-Infection. PLoS ONE.

[55] Fromentin R., Bakeman W., Lawani M.B., Khoury G., Hartogensis W., DaFonseca S., Killian M., Epling L., Hoh R., Sinclair E. (2016). CD4^+^ T Cells Expressing PD-1, TIGIT and LAG-3 Contribute to HIV Persistence during ART. PLoS Pathog..

[56] Sada-Ovalle I., Ocaña-Guzman R., Pérez-Patrigeón S., Chávez-Galán L., Sierra-Madero J., Torre-Bouscoulet L., Addo M.M. (2015). Tim-3 blocking rescue macrophage and T cell function against *Mycobacterium tuberculosis* infection in HIV+ patients. J. Int. AIDS Soc..

[57] https://naco.gov.in/sites/default/files/NACP_V_Strategy_Booklet.pdf.

[58] Sambrook J., Fritsch E.F., Maniatis T. (1989). Molecular Cloning a Laboratory Manual.

[59] Fenwick C., Joo V., Jacquier P., Noto A., Banga R., Perreau M., Pantaleo G. (2019). T-cell exhaustion in HIV infection. Immunol. Rev..

[60] McLane L.M., Abdel-Hakeem M.S., Wherry E.J. (2019). CD8 T Cell Exhaustion During Chronic Viral Infection and Cancer. Annu. Rev. Immunol..

[61] Labuschagne Naidoo R.-B., Steel H.C., Theron A.J., Anderson R., Tintinger G.R., Rossouw T.M. (2024). Persistently Elevated Expression of Systemic, Soluble Co-Inhibitory Immune Checkpoint Molecules in People Living with HIV before and One Year after Antiretroviral Therapy. Pathogens.

[62] Gay C.L., Bosch R.J., Ritz J., Hataye J.M., Aga E., Tressler R.L., Mason S.W., Hwang C.K., Grasela D.M., Ray N. (2017). Clinical Trial of the Anti-PD-L1 Antibody BMS-936559 in HIV-1 Infected Participants on Suppressive Antiretroviral Therapy. J. Infect. Dis..

[63] Colston E., Grasela D., Gardiner D., Bucy R.P., Vakkalagadda B., Korman A.J., Lowy I. (2018). An open-label, multiple ascending dose study of the anti-CTLA-4 antibody ipilimumab in viremic HIV patients. PLoS ONE.

[64] Rasmussen T.A., Rajdev L., Rhodes A., Dantanarayana A., Tennakoon S., Chea S., Spelman T., Lensing S., Rutishauser R., Bakkour S. (2021). Impact of Anti-PD-1 and Anti-CTLA-4 on the Human Immunodeficiency Virus (HIV) Reservoir in People Living With HIV With Cancer on Antiretroviral Therapy: The AIDS Malignancy Consortium 095 Study. Clin. Infect. Dis..

[65] Lavole A., Mazieres J., Schneider S., Brosseau S., Kiakouama L.M., Greillier L., Guihot A., Abbar B., Baron M., Makinson A. (2020). 1389P IFCT-1602 CHIVA2 phase II trial: Nivolumab in previously treated HIV-patients with advanced non-small cell lung cancer (NSCLC). Ann. Oncol..

[66] Gonzalez-Cao M., Morán T., Dalmau J., Garcia-Corbacho J., Bracht J.W.P., Bernabe R., Juan O., de Castro J., Blanco R., Drozdowskyj A. (2020). Assessment of the Feasibility and Safety of Durvalumab for Treatment of Solid Tumors in Patients With HIV-1 Infection. JAMA Oncol..

[67] Toor J.S., Singh S., Sharma A., Arora S.K. (2014). *Mycobacterium tuberculosis* Modulates the Gene Interactions to Activate the HIV Replication and Faster Disease Progression in a Co-Infected Host. PLoS ONE.

[68] Waters R., Ndengane M., Abrahams M.-R., Diedrich C.R., Wilkinson R.J., Coussens A.K. (2020). The Mtb-HIV syndemic interaction: Why treating M. tuberculosis infection may be crucial for HIV-1 eradication. Future Virol..

[69] Collins K.R., Quiñones-Mateu M.E., Toossi Z., Arts E.J. (2002). Impact of tuberculosis on HIV-1 replication, diversity, and disease progression. AIDS Rev..

[70] Wong K., Nguyen J., Blair L., Banjanin M., Grewal B., Bowman S., Boyd H., Gerstner G., Cho H.J., Panfilov D. (2020). Pathogenesis of Human Immunodeficiency Virus-*Mycobacterium tuberculosis* Co-Infection. J. Clin. Med..

[71] Mehta G., Sharma A., Arora S.K. (2021). Human Immunodeficiency Virus-1 Subtype-C Genetically Diversify to Acquire Higher Replication Competence in Human Host with Comorbidities. AIDS Res. Hum. Retroviruses.

[72] Mehta G., Sharma A., Arora S.K. (2021). Short Communication: Acquisition of Additional Nuclear Factor Kappa B Binding Sites in Long Terminal Repeat of Genetically Evolving HIV-1 Subtype C Viral Species in Host with Comorbidities. AIDS Res. Hum. Retroviruses.

[73] Kedzierska K., Crowe S.M., Turville S., Cunningham A.L. (2003). The influence of cytokines, chemokines and their receptors on HIV-1 replication in monocytes and macrophages. Rev. Med. Virol..

[74] Briken V., Porcelli S.A., Besra G.S., Kremer L. (2004). Mycobacterial lipoarabinomannan and related lipoglycans: From biogenesis to modulation of the immune response. Mol. Microbiol..

[75] Suzuki Y., Suda T., Asada K., Miwa S., Suzuki M., Fujie M., Furuhashi K., Nakamura Y., Inui N., Shirai T. (2012). Serum Indoleamine 2,3-Dioxygenase Activity Predicts Prognosis of Pulmonary Tuberculosis. Clin. Vaccine Immunol..

[76] Neogi U., Bontell I., Shet A., Costa A.D., Gupta S., Diwan V., Laishram R.S., Wanchu A., Ranga U., Banerjea A.C. (2012). Molecular Epidemiology of HIV-1 Subtypes in India: Origin and Evolutionary History of the Predominant Subtype, C. PLoS ONE.

[77] Seth P. (2010). Evolution of HIV-1 in India. Indian J. Virol..

[78] Holderried T.A.W., de Vos L., Bawden E.G., Vogt T.J., Dietrich J., Zarbl R., Bootz F., Kristiansen G., Brossart P., Landsberg J. (2019). Molecular and immune correlates of TIM-3 (HAVCR2) and galectin 9 (LGALS9) mRNA expression and DNA methylation in melanoma. Clin. EpiGenet..

[79] Chihara N., Madi A., Kondo T., Zhang H., Acharya N., Singer M., Nyman J., Marjanovic N.D., Kowalczyk M.S., Wang C. (2018). Induction and transcriptional regulation of the co-inhibitory gene module in T cells. Nature.

[80] Kgoadi K., Bajpai P., Ibegbu C.C., Dkhar H.K., Enriquez A.B., Dawa S., Cribbs S.K., Rengarajan J. (2025). Alveolar macrophages from persons with HIV mount impaired TNF signaling networks to M. tuberculosis infection. Nat. Commun..

[81] Shankar E.M., Saeidi A., Vignesh R., Velu V., Larsson M. (2018). Understanding Immune Senescence, Exhaustion, and Immune Activation in HIV–Tuberculosis Coinfection. Handbook of Immunosenescence.

[82] Chew G.M., Fujita T., Webb G.M., Burwitz B.J., Wu H.L., Reed J.S., Hammond K.B., Clayton K.L., Ishii N., Abdel-Mohsen M. (2016). TIGIT Marks Exhausted T Cells, Correlates with Disease Progression, and Serves as a Target for Immune Restoration in HIV and SIV Infection. PLoS Pathog..

[83] Moskophidis D., Lechner F., Pircher H., Zinkernagel R.M. (1993). Virus persistence in acutely infected immunocompetent mice by exhaustion of antiviral cytotoxic effector T cells. Nature.

[84] Zajac A.J., Blattman J.N., Murali-Krishna K., Sourdive D.J., Suresh M., Altman J.D., Ahmed R. (1998). Viral immune evasion due to persistence of activated T cells without effector function. J. Exp. Med..

[85] Gallimore A., Glithero A., Godkin A., Tissot A.C., Plückthun A., Elliott T., Hengartner H., Zinkernagel R. (1998). Induction and exhaustion of lymphocytic choriomeningitis virus-specific cytotoxic T lymphocytes visualized using soluble tetrameric major histocompatibility complex class I-peptide complexes. J. Exp. Med..

[86] Kahan S.M., Wherry E.J., Zajac A.J. (2015). T Cell Exhaustion During Persistent Viral Infections. Virology.

[87] Sakuishi K., Apetoh L., Sullivan J.M., Blazar B.R., Kuchroo V.K., Anderson A.C. (2010). Targeting Tim-3 and PD-1 pathways to reverse T cell exhaustion and restore anti-tumor immunity. J. Exp. Med..

[88] Jin H.-T., Anderson A.C., Tan W.G., West E.E., Ha S.-J., Araki K., Freeman G.J., Kuchroo V.K., Ahmed R. (2010). Cooperation of Tim-3 and PD-1 in CD8 T-cell exhaustion during chronic viral infection. Proc. Natl. Acad. Sci. USA.

[89] Liu J., Zhang S., Hu Y., Yang Z., Li J., Liu X., Deng L., Wang Y., Zhang X., Jiang T. (2016). Targeting PD-1 and Tim-3 Pathways to Reverse CD8 T-Cell Exhaustion and Enhance Ex Vivo T-Cell Responses to Autologous Dendritic/Tumor Vaccines. J. Immunother..

[90] Yadav R., Redmond W.L. (2022). Current Clinical Trial Landscape of OX40 Agonists. Curr. Oncol. Rep..

[91] Li J., Huang H.-H., Tu B., Zhou M.-J., Hu W., Fu Y.-L., Li X.-Y., Yang T., Song J.W., Fan X. (2021). Reversal of the CD8^+^ T-Cell Exhaustion Induced by Chronic HIV-1 Infection Through Combined Blockade of the Adenosine and PD-1 Pathways. Front. Immunol..

