# Supplementary material for: Mycobacterium tuberculosis Modulates the Expansion of Terminally Exhausted CD4+ and CD8+ T-Cells in Individuals with HIV-TB Co-Infection
Source: Pathogens. 2025 Aug 11;14(8):802. doi: 10.3390/pathogens14080802 (PMC12389345; doi:10.3390/pathogens14080802)
Supplement: Supplementary file 1 [file pathogens-14-00802-s001.zip › pathogens-3668346-supplementary.docx]

**Supplementary Table S1: Primer sequences, amplicon size and respective optimal annealing temperature for each Immune checkpoint molecules (ICMs) and housekeeping genes:**

| **Gene** | **Forward Primer (5’-3’)** | **Reverse Primer (5’-3’)** | **Amplicon size (bp)** | **Annealing Temp. (℃)** |
| --- | --- | --- | --- | --- |
| TIM-3 | TTGGACATCCAGATACTGGCT | CACTGTCTGCTAGAGTCACATTC | 86 bp | 60℃ |
| TIGIT | TGGTCGCGTTGACTAGAAAGA | GGGCTCCATTCCTCCTGTC | 94 bp | 60℃ |
| LAG-3 | GCGGGGACTTCTCGCTATG | GGCTCTGAGAGATCCTGGGG | 158 bp | 60℃ |
| CTLA-4 | CATGATGGGGAATGAGTTGACC | TCAGTCCTTGGATAGTGAGGTTC | 92 bp | 60℃ |
| PD-1 | CGTGGCCTATCCACTCCTCA | ATCCCTTGTCCCAGCCACTC | 106 bp | 60℃ |
| β-Actin | AAATCTGGCACCACACCTTC | GGGGTGTTGAAGGTCTCAAA | 139bp | 58℃ |

Abbreviations: Immune checkpoint molecules (ICMs), T-cell Immunoglobulin and Mucin-domain containing-3 (TIM-3), T-cell Immunoreceptor with Ig and immunoreceptor tyrosine-based inhibitory motif domains (TIGIT), Lymphocyte Activation Gene-3 (LAG-3), Cytotoxic T-Lymphocyte Antigen-4 (CTLA-4), Programmed Death-1 (PD-1), Beta-actin (β-Actin).

**
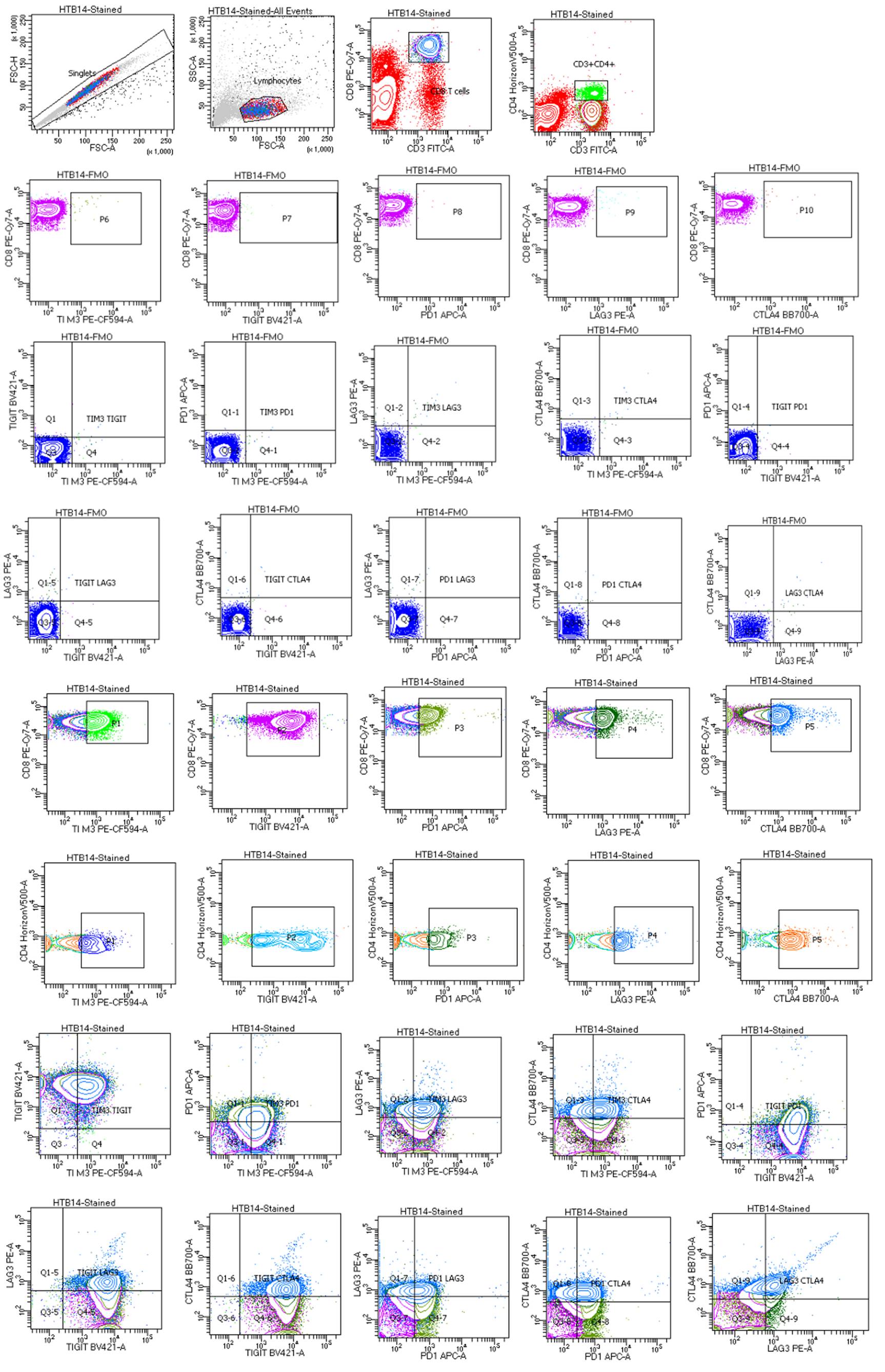
Supplementary Figure S1.** G**ating strategy used to identify CD4^+^ T helper and CD8^+^ cytotoxic T-lymphocytes expressing ICMs in human peripheral blood.** Antibodies that specifically recognize the cell-surface markers CD3, CD8, CD4, TIM-3, TIGIT, PD-1, LAG-3 and CTLA-4, and conjugated to the fluorescent dyes FITC, PE-Cy7, Horizon V500, PE-CF594, BV421, APC, PE and BB700 respectively. For every patient, we prepared three tubes to establish gating strategy and criteria for selecting positive events: Tube 1. Unstained cells (Cells alone tube): This tube was used to determine baseline auto fluorescence of the cells. Singlets were identified based on forward scatter area (FSC-A) versus forward scatter height (FSC-H) and lymphocytes were gated based on a forward scatter area (FSC-A) versus side scatter area (SSC-A) plot. Tube 2. FM5 (Fluorescence minus 5) tube (Referred as FMO tube): This tube contains cells labelled with antibodies against CD3, CD4 and CD8, while omitting the antibodies specific to any ICMs. This tube was used to identify CD4^+^ and CD8^+^ T-cell clusters and set the negative gates for each of ICMs. Tube 3. Stained tube: Cells labelled with CD3, CD4 and CD8 and five ICMs (TIM-3, TIGIT, PD-1, LAG-3 and CTLA-4): This tube was used to detect the expression of ICMs on CD4^+^ and CD8^+^ T-cells populations. The selection of positive events or the frequencies of CD4^+^ and CD8^+^ T-cells expressing ICMs were based on the negative gates set in Tube 2. The analysis included the expression of TIM-3, LAG-3, PD-1, CTLA-4, and TIGIT individually as well as in pairs: TIM-3^+^ TIGIT^+^, TIM-3^+^ PD-1^+^, TIM-3^+^ LAG-3^+^, TIM-3^+^ CTLA-4^+^, TIGIT^+^ PD-1^+^, TIGIT^+^ LAG-3^+^, TIGIT^+^ CTLA-4^+^, PD-1^+^ LAG-3^+^, PD-1^+^ CTLA-4^+^ and LAG-3^+^ CTLA-4^+^.


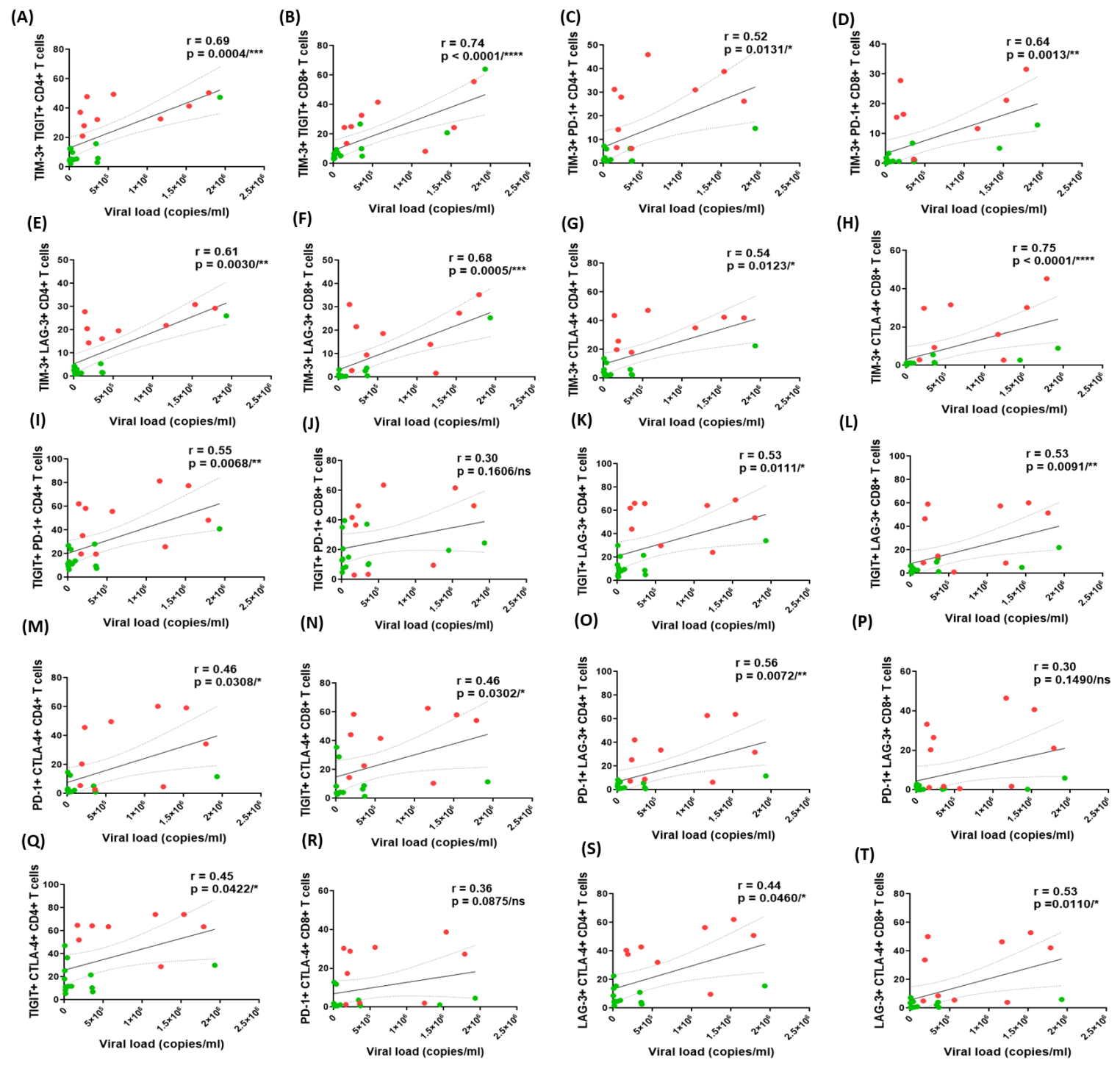


**Supplementary Figure S2.** **Correlation between the frequencies of CD4^+^ and CD8^+^ T cells co-expressing dual ICMs with the HIV-1 plasma viral load.** Correlations between the frequencies of CD4^+^ and CD8^+^ T cells co-expressing ICMs: TIM-3^+^ TIGIT^+^, TIM-3^+^ PD-1^+^, TIM-3^+^ LAG-3^+^, TIM-3^+^ CTLA-4^+^, TIGIT^+^ PD-1^+^, TIGIT^+^ LAG-3^+^, TIGIT^+^ CTLA-4^+^, PD-1^+^ LAG-3^+^, PD-1^+^ CTLA-4^+^ and LAG-3^+^ CTLA-4^+^ **(A-T**) with plasma viral load in recruited HIV mono infected and HIV-TB co-infected individuals. Red dots represent HIV-TB co-infected individuals and green dots represent HIV mono-infected individuals. Each dot represents one individual. The solid line represents the linear regression of points and dotted lines represents 95% confidence band for their mean values. r, spearman’s correlation coefficient. Level of significance, ns *p* ≥ 0.05, * *p* < 0.05, ** *p* < 0.01, *** *p* < 0.001 and **** *p* < 0.0001.

- The frequencies of TIM-3^+^ TIGIT^+^ CD4^+^ **(r = 0.69, *p =* 0.0004)** and TIM-3^+^ TIGIT^+^ CD8^+^ T cells **(r = 0.74, *p <* 0.0001)**; TIM-3^+^ PD-1^+^ CD4^+^ **(r = 0.52,** ***p =* 0.0131)** and TIM-3^+^ PD-1^+^ CD8^+^ T cells **(r = 0.64,** ***p =* 0.0013)**; TIM-3^+^ LAG-3^+^ CD4^+^ **(r = 0.61, *p =* 0.0030)** and TIM-3^+^ LAG-3^+^ CD8^+^ T cells **(r = 0.68,** ***p =* 0.0005)**; TIM-3^+^ CTLA-4^+^ CD4^+^ **(r= 0.54,** ***p =* 0.0123**) and TIM-3^+^ CTLA-4^+^ CD8^+^ T cells **(r = 0.75,** ***p <* 0.0001)**; TIGIT^+^ PD-1^+^ CD4^+^ **(r = 0.55**, ***p =* 0.0068)** and; TIGIT^+^ LAG-3^+^ CD4^+^ **(r = 0.53**, ***p =* 0.0111**) and TIGIT^+^ LAG-3^+^ CD8^+^ T cells **(r = 0.53,** ***p =* 0.0091)**; TIGIT^+^ CTLA-4^+^ CD4^+^ **(r = 0.45,** ***p =* 0.0422)** and TIGIT^+^ CTLA-4^+^ CD8^+^ T cells **(r = 0.46,** ***p =* 0.0302)**; PD-1^+^ LAG-3^+^ CD4^+^ **(r = 0.56,** ***p =* 0.0072)**; PD-1^+^ CTLA-4^+^ CD4^+^ **(r = 0.46, *p =* 0.0308)**; LAG-3^+^ CTLA-4^+^ CD4^+^ **(r = 0.44,** ***p =* 0.0460)** and LAG-3^+^ CTLA-4^+^ CD8^+^ **(r = 0.53, *p =* 0.0110)** in recruited HIV mono-infected and HIV-TB co-infected individuals were positively correlated with the Viral load **(Supplementary Figure S2 A-I, K-O, Q, S-T respectively).** However no correlation was observed between TIGIT^+^ PD-1^+^ CD8^+^ T cells **(r = 0.30,** ***p =* 0.1606),** PD-1^+^ CTLA-4^+^ CD8^+^ **(r = 0.30,** ***p =* 0.1490),** PD-1^+^ CTLA-4^+^ CD8^+^T cells **(r = 0.36,** ***p =* 0.0875)** **(Supplementary Figure S2 I,P,R respectively)** with the viral load.

**Supplementary Figure S3.** **Correlation between the frequencies of CD4^+^ and CD8^+^ T cells co-expressing dual ICMs with the absolute CD4^+^ T-cell counts.** Correlations between the frequencies of CD4^+^ and CD8^+^ T cells co-expressing ICMs: TIM-3^+^ TIGIT^+^, TIM-3^+^ PD-1^+^, TIM-3^+^ LAG-3^+^, TIM-3^+^ CTLA-4^+^, TIGIT^+^ PD-1^+^, TIGIT^+^ LAG-3^+^, TIGIT^+^ CTLA-4^+^, PD-1^+^ LAG-3^+^, PD-1^+^ CTLA-4^+^ and LAG-3^+^ CTLA-4^+^ **(A-T)** with the absolute CD4^+^ T cell count in recruited HIV mono infected and HIV-TB co-infected individuals. Red dots represent HIV-TB co-infected individuals and green dots represent HIV mono-infected individuals. Each dot represents one individual. The solid line represents the linear regression of points and dotted lines represents 95% confidence band for their mean values. r, spearman’s correlation coefficient. Level of significance, * *p* < 0.05, ** *p* < 0.01, *** *p* < 0.001 and **** *p*  < 0.0001.

- The frequencies of TIM-3^+^ TIGIT^+^ CD4^+^ **(r = −0.75,** ***p <* 0.0001)** and TIM-3^+^ TIGIT^+^ CD8^+^ T cells **(r = −0.56,** ***p =* 0.0068)**; TIM-3^+^ PD-1^+^ CD4^+^ **(r = −0.80, *p <* 0.0001)** and TIM-3^+^ PD-1^+^CD8^+^ T cells **(r = −0.69. *p =* 0.0002)**; TIM-3^+^ LAG-3^+^ CD4^+^ **(r = −0.71, *p =* 0.0001)** and TIM-3^+^ LAG-3^+^ CD8^+^ T cells **(r = −0.79, *p <* 0.0001)**; TIM-3^+^ CTLA-4^+^ CD4^+^ **(r = −0.59, *p =* 0.0024)** and TIM-3^+^ CTLA-4^+^ CD8^+^ T cells **(r = −0.77, *p* < 0.0001)**; TIGIT^+^ PD-1^+^ CD4^+^ **(r = −0.80,** ***p <* 0.0001**) and TIGIT^+^ PD-1^+^ CD8^+^ T cells **(r = −0.52, *p =* 0.0094)**; TIGIT^+^ LAG-3^+^ CD4^+^ **(r = −0.75, *p <* 0.0001)** and TIGIT^+^ LAG-3^+^ CD8^+^ T cells **(r = −0.59, *p =* 0.0032)**; TIGIT^+^ CTLA-4^+^ CD4^+^ **(r = −0.68, *p =* 0.0004)** and TIGIT^+^ CTLA-4^+^ CD8^+^ T cells **(r = −0.72, *p =* 0.0002)**; PD-1^+^ LAG-3^+^ CD4^+^ **(r = −0.74, *p <* 0.0001),** PD-1^+^ LAG-3^+^ CD8^+^ **(r = −0.52, *p =* 0.0103)**; PD-1^+^ CTLA-4^+^ CD4^+^ **(r = −0.73, *p <* 0.0001)** and PD-1^+^ CTLA-4^+^ CD8^+^ **(r = −0.70, *p =* 0.0002)**; LAG-3^+^ CTLA-4^+^ CD4^+^ **(r = −0.67, *p =* 0.0004)** and LAG-3^+^ CTLA-4^+^ CD8^+^ **(r= −0.77, *p <* 0.0001)** in recruited HIV mono infected and HIV-TB co-infected individuals were negatively correlated with the absolute CD4^+^ T cell count. **(Supplementary Figure S3 A-T respectively).**
